# Classroom Integration of AI-Simulated Patients and Automated Feedback in Doctoral Psychotherapy Training: Longitudinal Mixed Methods Study

**DOI:** 10.2196/93199

**Published:** 2026-11-11

**Authors:** Ellen Converse, Ryan Louie, Debra Safer, Juan Pablo Pacheco, William Fang, Jamie Kent, Bruce Arnow, Diyi Yang

**Affiliations:** 1 PAU-Stanford PsyD Consortium, University Palo Alto, 1791 Arastradero Road, Palo Alto, CA, 94304, United States, 1 650-498-9111; 2 Department of Computer Science, Stanford University, 353 Jane Stanford Way, Stanford, CA, 94305, United States; 3 Department of Psychiatry and Behavioral Sciences, Stanford University School of Medicine, 401 Quarry Road, Stanford, CA, 94304, United States

**Keywords:** artificial intelligence, psychotherapy, deliberate practice, classroom deployment, AI-simulated patients

## Abstract

**Background:**

Psychotherapy education increasingly emphasizes technology-assisted methods that extend deliberate practice beyond human supervision. AI has enabled automated skill assessment and patient simulations, yet few studies have examined how students experience such tools in real-world settings alongside established practice modalities.

**Objective:**

We aimed to evaluate students’ experiences with AI-augmented deliberate practice embedded in an existing psychotherapy course sequence. Secondary aims included identifying implementation barriers, ethical and pedagogical considerations, and factors influencing students’ acceptance of AI-assisted learning.

**Methods:**

Participants were 29 first-year clinical psychology doctoral students in a 2-quarter psychotherapy sequence at an American Psychological Association–accredited program. In the fall course, students completed weekly peer role-plays and could optionally submit transcripts to CARE for AI feedback to supplement peer feedback. In the winter course, students completed required weekly deliberate practice through Skillsetter video vignettes and could optionally practice with CARE’s voice-based AI-simulated patients, receiving AI and instructor feedback afterward. Surveys with Likert-type and free-response items were voluntary in the fall and required in the winter; 9 and 25 students completed them, respectively. Analyses included descriptive statistics, matched-pairs testing, and thematic analysis.

**Results:**

In the fall course (n=9), where privacy concerns and survey nonresponse limited data on AI feedback, students rated peer feedback higher than AI feedback on being constructive and helpful (median 3, IQR 3-3 vs median 2, IQR 2-2; *P*=.02) and recognizing areas to improve (mean 5.22, SD 0.83 vs mean 4.33, SD 0.87; *P*=.046), citing transcription errors and a narrow focus on empathy while valuing AI’s alternative phrasing. In the winter course (n=25), Skillsetter was rated higher than CARE only for learning from mistakes (mean 4.52, SD 2.00 vs mean 3.20, SD 2.04; *P*=.03); immersion, spontaneous responding, communication beyond words, and alliance building did not differ significantly. Students valued CARE for interactive practice not possible with vignettes but reported undetected vocal pauses (20/25, 80%), insufficient realism (14/25, 56%), and limited emotional expression (13/25, 52%), and raised concerns about AI replacing peer support (22/25, 88%) and peer interaction (20/25, 80%). Asked about AI broadly, students expressed mixed views, citing concerns about supplanted relational dynamics, nonconsensual data use, and generative AI’s environmental impacts.

**Conclusions:**

Embedding AI-augmented deliberate practice into an existing course sequence was feasible, but students’ experiences favored established methods: they preferred human over AI feedback and described video vignettes as more realistic than voice-based AI interactions, while valuing AI-simulated patients for interactive practice other modalities could not provide. Implementation barriers included feedback quality, transcription accuracy, and instructor time required to supplement AI feedback. Perceptions of AI’s instructional utility appeared contingent on students’ trust in the technology, suggesting future deployments should weigh a sample’s attitudes toward AI and its value relative to existing training resources.

## Introduction

### Background

Psychotherapy is a structured clinical intervention wherein trained professionals use established psychological methods, primarily through dialogue and relational engagement, to alleviate distress, reduce psychiatric symptoms, and promote psychological functioning and well-being [1,2]. Psychotherapy is a collaborative process in which the therapeutic alliance, defined as the emotional bond between the therapist and patient, as well as agreement on the goals of therapy and the tasks required to achieve the therapeutic goals [3], is consistently associated with clinical improvement [4].

Therapeutic communication skills cannot be fully developed through lectures or reading alone; instead, they are cultivated through experiential practice, structured feedback, and critical reflection. Accordingly, traditional psychotherapy training emphasizes didactic instruction (structured, instructor-led teaching methods, such as lectures, seminars, or presentations that convey foundational theories and practical guidelines) alongside supervised clinical practice, role-play, and reflective exercises. Collectively, these methods facilitate real-time interaction and iterative refinement of core therapeutic skills [5].

Training psychotherapists, therefore, presents a distinctive pedagogical challenge: students must master not only theoretical and technical knowledge but also deeply interpersonal competencies, including empathic attunement, alliance building, and moment-to-moment responsiveness. A small yet steadily growing evidence base suggests that deliberate practice (DP) is an effective learning method that can outperform standard supervision and training [6]. Unlike didactic instruction, DP focuses on structured and intentional skill development through activities that include clearly defined goals, repeated practice, systematic opportunities for reflection, and real-time feedback from a supervisor or coach.

A common form of DP in graduate psychotherapy training contexts consists of a real-time session with a peer or clinical supervisor role-playing a simulated scenario and/or providing feedback following a rehearsal or therapy recording. Empirical evidence supports the effectiveness of DP in enhancing these skills. For example, learners who engaged in DP showed greater improvements in empathic expression compared to those who were trained using didactic methods alone [7]. Despite its potential benefits, the widespread implementation of DP in clinical psychology training programs is limited by the significant time and expertise required for 1-on-1, real-time supervision, and personalized feedback [6].

### Technological Innovations in Psychotherapy Training

Researchers have sought to enhance the accessibility and efficiency of psychotherapy training by developing technology-enabled platforms. Early efforts focused on structured tools for DP, such as video recording systems, which facilitate asynchronous supervision and reflective practice by enabling both students and instructors to review therapist-patient interactions outside of real-time sessions [8]. Similarly, video-response platforms such as Skillsetter (Theravue Inc) [9] offer structured opportunities for practice by presenting short patient vignettes and requiring trainees to record therapeutic responses to those vignettes, which can later be reviewed for feedback by peers or supervisors. These innovations have expanded opportunities for repeated skill rehearsal, supplementing traditional classroom and supervisory contexts.

Recent advances in large language models (LLMs) have further broadened this landscape, particularly through the development of AI-simulated patients that allow counselors to engage in practice dialogues within safe, controlled environments [10,11]. Parallel work has advanced AI feedback systems capable of automatically assessing microskills such as empathy, active listening, and motivational interviewing [12-16], while also generating alternative responses [8,17,18] and explanatory rationales [19,20].

Within the emerging literature, AI-augmented psychotherapy training systems show both potential and methodological limitations. Recent experimental laboratory studies suggest that such simulations, when paired with feedback, may promote the acquisition of patient-centered counseling behaviors [21,22]. Avatar-based simulated patients can effectively support motivational interviewing practice, garnering high levels of usability and satisfaction among both student and professional counselors [23]. More recently, AI-simulated patients have been assigned within clinical education settings such as psychotherapy [24,25] and medical education [26]. However, even the most promising implementations fall short of assessing how trainees engage with AI over extended periods alongside traditional coursework, supervision, and peer practice. Consequently, questions remain about how trainees engage with AI tools when integrated into coursework as part of ongoing real-world classroom settings rather than deployed as standalone exercises.

### The Current Investigation

Addressing the limitations of prior work, this study seeks to advance the understanding of how AI-augmented DP functions when directly embedded into psychotherapy training. As reviewed earlier, existing research on AI tools has primarily been restricted to controlled laboratory settings, short intervention windows, or isolated evaluations conducted outside of the classroom context. Such studies cannot fully address how AI interacts with real pedagogical structures, classroom dynamics, or established training methods such as peer role-play, supervised feedback, and video vignette exercises. Moreover, it remains unclear whether AI practice tools operate as meaningful complements to these traditional approaches, whether they inadvertently displace more valuable training modalities, or whether their integration reshapes learning in unanticipated ways.

To address these gaps, the present investigation examined students’ experiences with CARE, a voice-based, AI-augmented DP platform on which users speak in real time with AI-simulated patients, mirroring the pacing and unpredictability of an actual patient encounter rather than clicking through scripted prompts, when deployed across 2 academic quarters within a doctoral-level psychotherapy curriculum. CARE was integrated alongside existing training methods, including peer role-plays and the Skillsetter video-response platform, to enable structured comparison of AI-based approaches with established educational tools. The primary objective of the study was to evaluate the feasibility and perceived value of implementing CARE in this classroom context. Secondarily, we explored implementation barriers, ethical and pedagogical considerations, and factors influencing students’ acceptance of and engagement with AI-assisted learning. Guided by these objectives, the study addressed 4 research questions (RQs):

- RQ1: Comparative value—From the perspective of students, how do 2 bundled DP packages, an AI-augmented package (CARE: voice-based, multiturn practice with AI feedback) and an established video-based package (Skillsetter: single-turn vignette practice with instructor feedback), compare in perceived instructional value, and what distinct affordances do students attribute to each package as delivered?
- RQ2: Usability and technical limitations—What factors about the AI-augmented package prototype (CARE) might enhance the pedagogical effectiveness of CARE within psychotherapy training?
- RQ3: Course integration influence on acceptability—How are utility and acceptability influenced by how the AI-augmented package (CARE) is embedded into the psychotherapy classroom, including issues of onboarding, communication, and strategies to preserve instructor-student and peer-to-peer interaction within the deployment?
- RQ4: Student beliefs about acceptance—(1) How do students perceive the legitimacy and acceptability of the AI-augmented package (CARE) in psychotherapy education, and (2) how has their exposure to AI in general influenced their views on its potential role in clinical training and practice?

Through its design, to our knowledge, the study provides one of the first longitudinal, classroom-based evaluations of an AI-augmented DP tool in psychotherapy education. By situating AI within authentic curricular structures rather than laboratory simulations, this work offers an ecologically valid assessment of both the pedagogical potential and the limitations of AI integration, thereby informing future directions for technology-mediated training in the mental health professions.

## Methods

### Ethical Considerations

The protocol for this psychotherapy classroom deployment study was reviewed by Stanford University Institutional Review Board and was assessed as nonmedical exempt (protocol ID 72546). This research was conducted as part of normal educational practice in an introductory psychotherapy course in a clinical psychology PsyD program at the Palo Alto University–Stanford PsyD Consortium. AI-augmented practice components were added to standard weekly assignments. The study included mid-class and end-class surveys to assess students’ perceptions of AI feedback compared to peer feedback. Students received an information sheet explaining that while participation in course activities was expected as part of normal educational practice, they could opt out of having their data included in research analysis by emailing the protocol director. The instructor had no knowledge of which students opted out, ensuring no coercion or impact on grades. No compensation was provided as the AI-augmented assignments and surveys were integrated into normal course requirements.

To protect privacy and confidentiality, all data transmission was encrypted via HTTPS and stored on password-protected Amazon Web Services (AWS) servers accessible only to investigators listed on the protocol. Survey responses were linked to deidentified participant IDs through Qualtrics (Qualtrics International Inc). The research team committed that practice transcripts would not be publicly released or used to train autonomous AI therapy chatbots; we further describe in *Data Privacy Protection in CARE’s System Architecture* section how we honor that commitment within our data and AI processing pipelines. Audio recordings and transcripts would be stored for up to 3 years to allow for the completion of all scientific analyses by the authors before deletion. Only deidentified survey data, aggregated usage statistics, and feedback ratings could be presented at scientific meetings or published in journals. The AI feedback system underwent safety validations prior to deployment, including guard models to filter inappropriate content.

### Participants

Participants were 29 first-year doctoral students enrolled in the abovementioned PsyD program. Of these participants, 4 students elected not to engage with CARE at any point during the study. The remaining 25 students participated in at least some component of the CARE activities and comprise the analytic sample reported here.

Students entered with heterogeneous training backgrounds: some held master’s degrees and had accumulated significant supervised clinical hours, whereas others entered with bachelor’s degrees supplemented by applied fieldwork or human services experience. This requirement for prior clinical experience differentiates PsyD training from many master’s mental health programs and some PhD programs, which often emphasize research over early clinical immersion.

Demographic data (eg, age, gender, and ethnicity) were not collected, which limits analysis of subgroup variation.

### Pedagogical Context and Course Goals

The study was embedded within a 2-quarter sequence forming part of the core first-year PsyD curriculum. This sequence is designed to provide foundational training in psychotherapy competencies, with a structured progression from introductory relational skills to more advanced therapeutic intervention. The study was conducted over 16 weeks, spanning 2 consecutive courses (September 2024 to March 2025).

The first course study emphasized pantheoretical elements of psychotherapy, including basic listening skills, therapeutic alliance formation, therapist verbal response models and intentions, and consideration of cultural and ethical factors. The second course quarter built upon these foundations by targeting pantheoretical competencies outlined by the American Psychological Association (APA) Division 29 Task Force on Psychotherapy Competence, such as alliance maintenance (task, goal, and bond), rupture repair, transference and countertransference, and termination processes.

### Historical Structure of the Learning Psychotherapy Course Series

In prior cohorts, the first course used weekly peer roleplays. Students alternated therapist and patient roles in 10-minute sessions, followed by mutual feedback and written self-reflections. Confidentiality agreements were required, and each student submitted recorded sessions that were periodically reviewed in small group meetings with instructors.

In the second course, prior cohorts engaged with Skillsetter’s video-based DP platform. Assignments required demonstration of specific therapeutic skills (eg, empathy, cultural humility, and alliance repair). Instructors or teaching assistants (TAs) provided feedback on a weekly basis, and the quarter culminated in a live roleplay with an instructor or TA portraying a patient, requiring integration of multiple therapeutic competencies. This established format of peer roleplay in the first course and Skillsetter video-based DP in the second course provided both relationally grounded and structured opportunities for iterative skill refinement.

### Skillsetter Platform

Skillsetter is a web-based, video-based DP training platform designed to support practice in interpersonal and counseling skills [27]. It was developed to overcome logistical barriers commonly encountered in traditional psychotherapeutic training, such as the resource-intensive nature of instructor supervision and inconsistent feedback, by allowing learners to practice asynchronously, record responses to simulated prompts, and receive structured feedback. When using the platform, students submitted video-recorded responses to standardized patient vignettes performed by actors. Because feedback, rather than mere repetition, is central to DP, Skillsetter emphasizes scaffolded evaluation, either via instructor raters or automated scoring across domains.

The platform has achieved broad adoption across counseling, social work, and psychotherapy training programs, lending evidence of its acceptability and institutional integration. In the Learning Psychotherapy course series, feedback on Skillsetter practice was given by the course instructor and TAs. In applying it to this study, Skillsetter served as an established, scalable platform for implementing component-level practice (eg, empathy, reflections, and open questions) in a remote environment. This enabled learners to engage in deliberate rehearsal outside class hours while receiving targeted feedback.

### Integration of CARE

Building on this structure, this study introduced CARE, an AI-powered simulation and feedback platform, to examine its potential role alongside traditional DP (Figure 1). Our design maintained the integrity of the existing pedagogy while creating opportunities for systematic comparison.

In the first course pilot study in 2024, the instructional team prioritized interpersonal skill building and peer connection, continuing to rely on peer role-play as the primary experiential method. CARE was introduced as an optional feedback tool, allowing students to upload recorded peer sessions and receive automated feedback (Figure 2) on the use of 8 counseling skills (eg, empathy, validation, reflections, questions, suggestions, self-disclosure, session structure, and professionalism) derived from the helping skills and motivational interviewing models [28,29]. This integration provided an opportunity to explore the additive value of AI-generated feedback while preserving the emphasis on human relational practice in the early stages of training.

**Figure 1.**
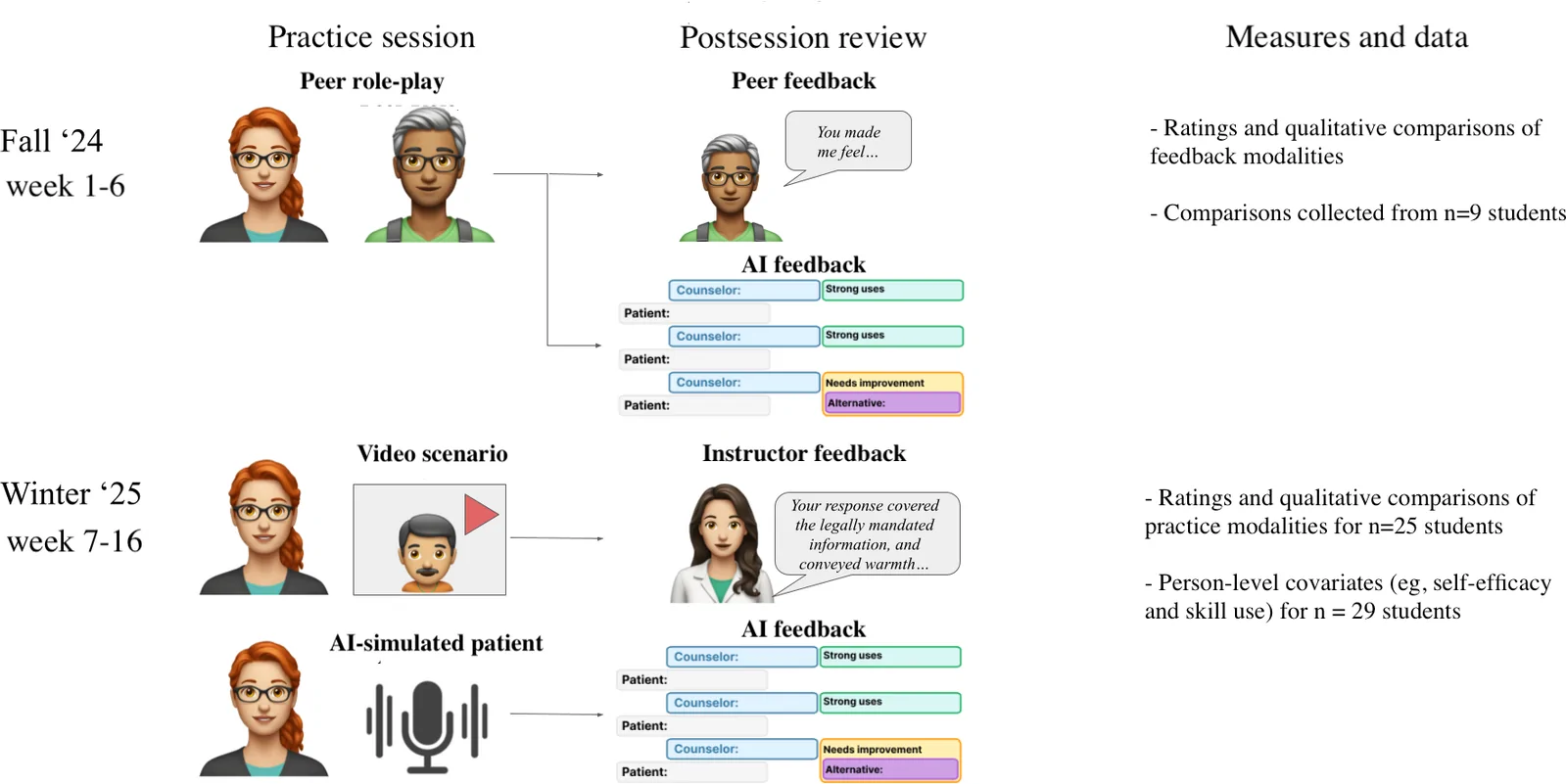
Overview of the integration of CARE’s practice, feedback, and data collection into the 2 courses. The first course implementation lasted 6 weeks, and the second course lasted 10 weeks. During the first course (weeks 1‐6), students engaged in peer role-play practice sessions followed by post-session review using either peer-provided or AI-generated feedback, with ratings and qualitative comparisons collected (n=9). During the second course (weeks 7‐16), students completed practice using video-based scenarios and AI-simulated patients, followed by instructor or AI-generated feedback, with ratings, qualitative comparisons, and person-level covariates (eg, self-efficacy and skill use) collected (n=25). The figure illustrates the progression of practice modalities, feedback sources, and data collection across the 2 courses.

**Figure 2.**
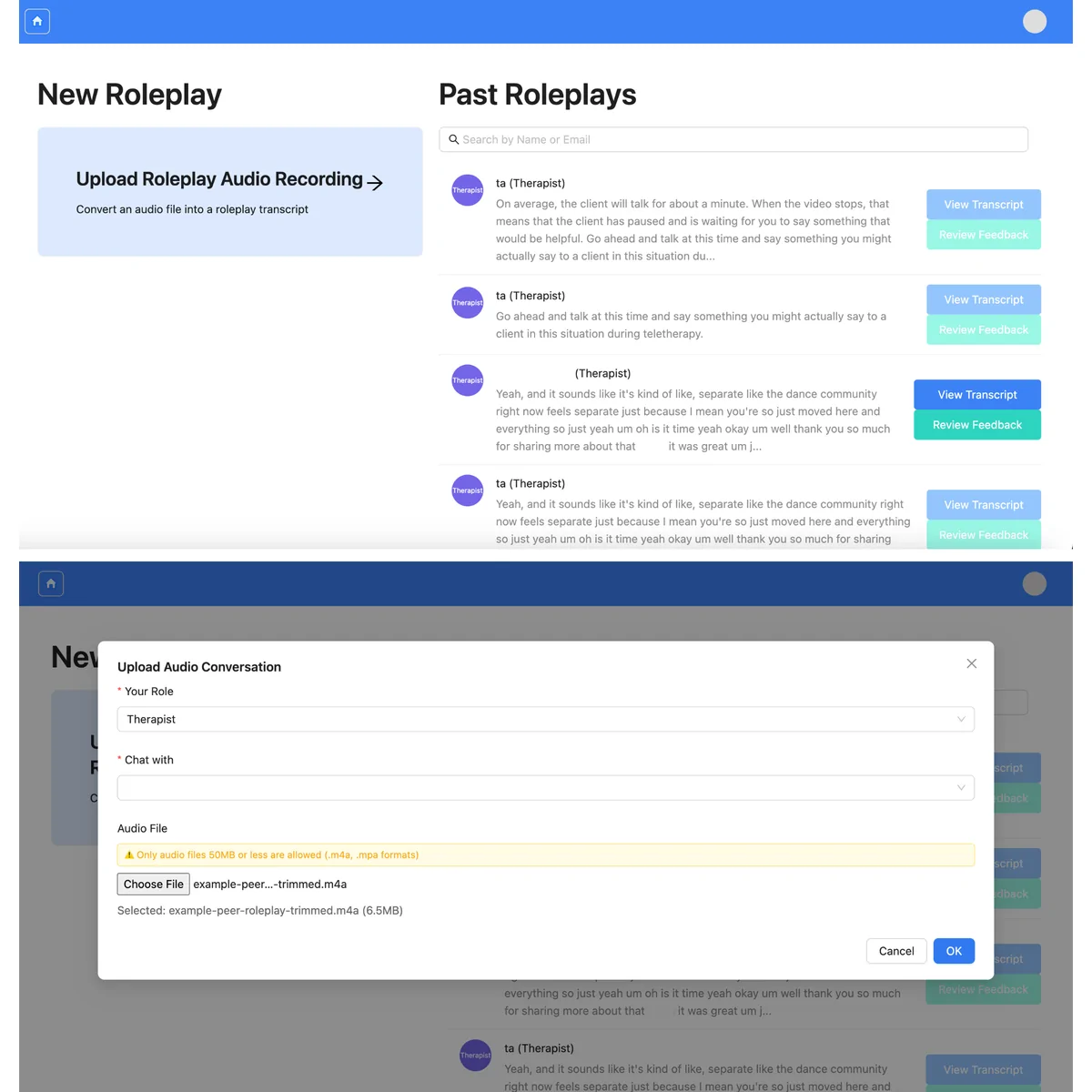
Workflow for AI feedback on peer role-plays in the first course. Students completed weekly peer-to-peer role-plays, alternating between therapist and patient roles, and recorded each session. Audio files uploaded to CARE were transcribed and diarized into speaker turns, and the platform generated feedback on the student’s utterances as therapist. The content shown is illustrative example data from test accounts; no student data are displayed.

In the second course (CLIN 702), CARE was deployed more comprehensively in parallel with Skillsetter to enable structured comparison of 2 distinct DP modalities (Table 1). Students alternated between Skillsetter’s single-turn vignette responses and CARE’s multiturn, dynamic dialogues with AI-simulated patients. Both platforms were aligned with weekly curricular objectives, matched for duration and credit, and graded using consistent rubrics. This dual-modality design created an ecologically valid setting for evaluating the relative affordances and limitations of AI-augmented practice versus established methods.

**Table 1.** Comparison of the 2 bundled deliberate practice packages evaluated in course 2^a^.

Dimension	CARE: voice-based AI-augmented package	Skillsetter: video-based package
Practice format	Multiturn, dynamic spoken dialogue with an LLM^b^-driven AI-simulated patient (voice; GPT-4o Realtime API)	Single-turn video-vignette response: 1 recorded reply to a standardized patient video performed by an actor
Feedback source	Automated AI feedback on each utterance (fine-tuned Llama 2 13B “mentor”); supplemented midquarter by weekly written TA^c^ feedback to 3 randomly selected students who completed a practice session	Human feedback from the course instructor and teaching assistants, using established rubric-based scoring
Number of sessions	10 weeks, 2 to 4 simulated patients offered as options per week	10 weeks, 1 simulated patient per week
Scenario type	AI-simulated patients spanning alliance ruptures, mandated reporting, and suicide risk/self-injury adapted from Skillsetter transcripts (9/12) and documented cases of sexually inappropriate behavior (3/12)	Standardized, actor-performed vignettes targeting specific skills (eg, empathy, cultural humility, and alliance repair)
Required versus optional	Initially required but was made optional in the second week. Students could complete additional Skillsetter practice instead of CARE	Established, required component of the course
Duration	Matched to Skillsetter for time and credit; weekly across weeks 7 to 16 (1‐2 minutes per session)	Weekly across weeks 7 to 16 (1‐2 minutes per session)
Grading	Rubrics matched to Skillsetter for evaluation criteria and grading weight	Rubric-based grading; quarter culminated in a live role-play via videoconference with an instructor or TA portraying the patient

a Course 1 involved a different comparison (AI-augmented feedback and peer feedback on peer role-plays) and is described separately.

b LLM: large language model.

c TA: teaching assistant.

### Safety Procedures for Emotionally Sensitive Scenarios

CARE’s scenario library included clinically challenging content, including alliance ruptures, mandated reporting, suicidal ideation and self-injury, and sexually inappropriate behavior toward clinicians. These topics were not introduced by the study. They are established components of the course curriculum, which addresses suicide risk and mandated reporting in week 7 and sexual attraction and boundary maintenance in week 8, and they reflect competencies specified by the APA Division 29 Task Force on Psychotherapy Competence [30]. Exposure to such material under supervision is a required element of doctoral clinical training, and these topics had been routinely taught in this course sequence with prior cohorts. CARE scenarios were mapped onto the existing weekly syllabus rather than developed independently, so students encountered simulated versions of material they were concurrently studying in class and practicing in Skillsetter.

Before deployment, each AI-simulated patient was reviewed by the TA and co-first author (EC), who had previously completed this course and held clinical training. Scenarios were additionally reviewed by the course instructor (JK), a licensed clinical psychologist and co-associate director of clinical training for the program, who confirmed their clinical appropriateness and alignment with the week’s learning objectives before they were released to students. Review criteria were clinical appropriateness, ethical soundness, and alignment with the week’s learning objectives. For scenarios involving trauma, self-harm, or suicidal ideation, review specifically assessed whether the AI-simulated patients supported rather than undermined the learning objective in the curriculum. Behavioral principles constraining each simulated patient were revised iteratively during this process. Nine of the 12 scenarios were adapted from existing Skillsetter transcripts already in curricular use; the remaining 3 were adapted from documented cases in the published literature [31].

Faculty oversight operated through the courses’ existing structures rather than through study-specific monitoring. Students had established channels for raising concerns: in-class discussion, direct contact with the instructor or TA, and the program’s existing student support structures. As the instructor and TA reviewed Skillsetter submissions on the same weekly topics, distress surfacing in either modality could be identified and addressed through the same channels.

We did not review CARE session transcripts in real time, and no automated system flagged student distress during practice. Safeguards were therefore preventive rather than responsive: scenario prompts were constrained in advance, and guard models filtered inappropriate simulated-patient output before students encountered it. No students reported clinically inappropriate or harmful content from the simulated patients. However, because we did not systematically review sessions, we cannot confirm that none occurred. We address this further in the “Limitations” section.

### CARE AI System

CARE was developed as a web-based training platform designed to support novice counselors in practicing counseling skills within simulated environments powered by LLMs. The platform enables trainees to practice psychotherapy skills through roleplay conversations either with their peers or with AI patients and to receive automated feedback on their therapeutic responses, generated by an AI “mentor.” CARE builds on prior work in co-design with mental health experts to enhance the realism of AI-simulated patients [8,10,11,26] and on research advancing the fine-tuning of domain-specific LLMs to incorporate therapeutic knowledge capable of producing both evaluative feedback and alternative counselor responses [6,19,32-34].

From a technical standpoint, CARE was implemented as a web application with a Python Flask (Pallets) back end and a React (Meta) JavaScript front end, accessible to students through password-protected student accounts. In the first course, the CARE platform allowed students to upload an audio recording of a peer role-play conversation, which was automatically converted into a diarized transcript. The transcription process used the WhisperX system (open source) and the OpenAI API to help with LLM-assisted diarization between therapist and patient utterances. In the second course, students practiced a role-play conversation with AI-simulated patients. AI-simulated patients were powered by the GPT-4o Realtime API, with scenario-specific prompts defining patient background, conversational style, and behavioral principles [10]. The OpenAI Whisper model was also used to transcribe the audio dialogue into text-based utterances. This infrastructure enabled integration into classroom deployment studies, allowing systematic investigation of CARE as a pedagogical supplement to established DP methods.

Within the version of CARE deployed to the second course, practice was organized around a library of patient scenarios, each defined by a brief background vignette (eg, “Young adult with family issues: low mood and self-esteem”). These intentionally limited descriptions are designed to approximate authentic counseling encounters, in which the clinician must simultaneously build rapport, elicit patient context, and apply therapeutic skills (Figure 3). For example, a novice peer counselor might initiate a practice session with an AI-simulated patient modeled as a 35-year-old veteran struggling to reconnect with his children amid legal and interpersonal challenges. The dialogue proceeds turn by turn, requiring the trainee to apply core counseling skills such as empathy, reflection, and problem exploration, while managing relational complexity as the conversation unfolds.

The platform provided students with AI-generated feedback on each of their utterances at the end of the session. CARE’s feedback implements an existing method for generating feedback that fine-tunes a Llama 2 13B (Meta) parameter model on an expert-annotated dataset covering psychotherapy-relevant skill assessment [19]. Feedback focuses on strengths, areas for improvement, feedback explanation, and a suggested alternative response that addresses any issues.

**Figure 3.**
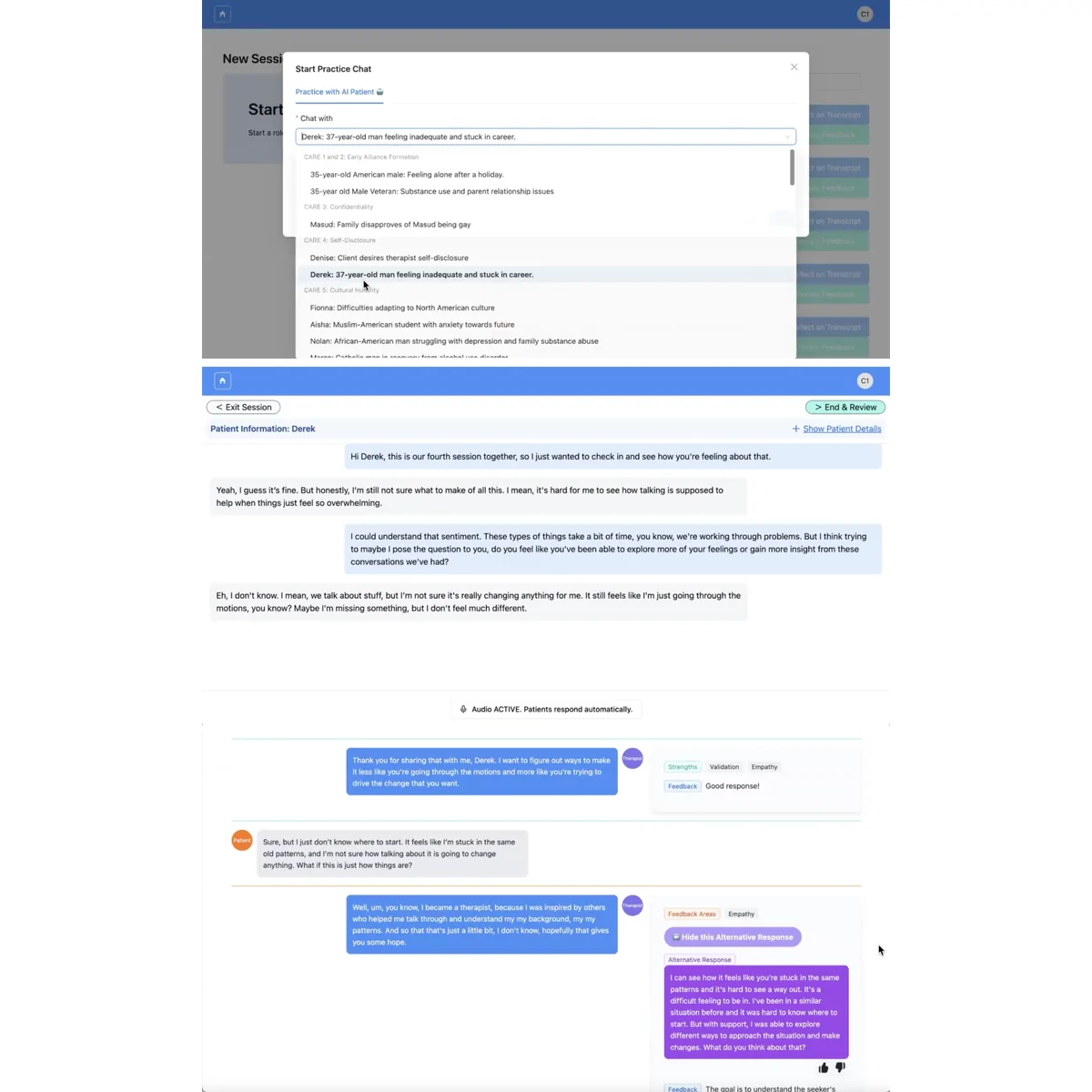
Weekly practice with AI-simulated patients in the second course. CARE offered students a set of voice-based AI-simulated patient scenarios each week, mapped onto that week’s learning goals (eg, therapist self-disclosure and cultural humility). Following each practice session, the platform generated feedback on the student’s utterances as therapist. The content shown is illustrative example data from test accounts; no student data are displayed.

### Data Privacy Protection in CARE’s System Architecture

CARE’s system architecture was designed to protect the privacy of student audio recordings and voice data and ensure they were not used for training AI models. In places where OpenAI APIs were used, the data policy was set to opt out of using any input or output data for AI model training; the OpenAI data retention policy further states that only abuse monitoring logs are retained for a maximum of 30 days.

For the pipeline that transcribed student peer role-play audio (course 1), uploaded audio files were saved onto the private AWS EC2 instance; audio was transported to an ephemeral, serverless graphics processing unit (GPU) service that started the WhisperX transcription and diarization function; the transcript was given to a GPT-4o-assisted diarization pipeline to identify therapist and patient roles; finally, the therapist/patient messages were saved to a SQLite database on the AWS EC2 instance. In this way, long-term data are only retained on our private AWS EC2 instance and are governed by our maximum 3-year retention policy to support scientific analysis.

The pipeline to practice with voice-based AI-simulated patients (course 2) is fully handled by the gpt-realtime API with authentication to OpenAI handled through ephemeral client API keys issued by the server. The gpt-realtime API end point responds to audio events and uses the OpenAI Whisper API to transcribe the audio transcript. Our OpenAI data privacy policy restricts long-term data retention and training on data sent to the API.

The feedback generation pipeline relies on an ephemeral, serverless GPU service that loads the fine-tuned Llama 2 13B model weights and processes the utterances in the transcript. The generated feedback is saved into the SQLite database. Thus, transcript and feedback data are only retained on the private AWS EC2 instance and are governed by our maximum 3-year data retention policy to allow for scientific analyses.

### Integration of CARE Into the Learning Psychotherapy Course Series

#### Overview

The Learning Psychotherapy sequence spanned 2 quarters, 16 weeks total, and was structured to provide developmentally sequenced training in core psychotherapy competencies. The first course implementation spanned 6 weeks, whereas the second course implementation extended across 10 weeks. The study team began the first course in week 4 of the first course instead of week 1 to allow more time for finalizing implementation procedures.

The first course 2024 pilot, described in more detail in the following section, served as an initial, formative phase of the study, designed to explore the educational utility of AI-generated feedback with traditional peer feedback following role-play exercises. This phase was crucial in identifying themes that shaped the subsequent second course study, including the relative value of different feedback modalities (RQ1), early design limitations (RQ2), challenges of classroom integration (RQ3), and students’ broader attitudes about the role of AI in psychotherapy training and daily life (RQ4).

#### First Course: Foundational Skills and Peer Role-Play

The first course provides a foundation for training beginning psychotherapists in the crucial elements of psychotherapy. Across weeks 1 to 7, instruction progressed via the gradual introduction of the core listening skills and verbal response modes, with particular attention in week 4 to how sexual, gender, and racial diversity can impact the therapeutic alliance. Weeks 8 to 9 introduced principles and strategies from motivational interviewing, with the course concluding in week 10 with a focus on ethics, professional boundaries, and therapist self-care.

Students engaged in 10-minute peer role-plays each week, alternating between therapist and patient roles, with assigned therapeutic skills tailored to that week’s topic. Recordings of these peer role-plays were uploaded to CARE, which generated automated feedback across 8 core counseling skill domains derived from the helping skills and motivational interviewing models. This process enabled the instructional team to evaluate student perceptions of AI-generated feedback as a supplement to traditional experiential exercises, while maintaining the central role of in-person peer practice in early clinical training.

#### Second Course: Comparative Deployment of Skillsetter and CARE

The second course introduced students to the principles of DP, values-based care, and basic therapeutic skills. Instruction progressed from the concept of DP (week 1) to the therapeutic alliance, drawing on Bordin’s task-goal-bond framework (week 2) [35], before moving to therapist self-disclosure, cultural humility, and the influence of power and identity within therapeutic relationships (weeks 3 and 4). The middle phase emphasized countertransference, resistance (week 5), alliance ruptures and repair (week 6), and clinical challenges such as mandated reporting and suicide risk (week 7). The sequence concluded with advanced relational concerns, including sexual attraction and boundary maintenance (week 8).

The course was designed to directly compare 2 distinct DP modalities: Skillsetter and CARE. This parallel design created a side-by-side comparison of vignette-based practice and conversational AI simulations, with both modalities aligned to weekly curricular goals and equivalent in duration, evaluation criteria, and grading weight.

To integrate CARE into the curriculum, the study team systematically mapped AI-simulated patients onto weekly course topics at week 4, enabling students to engage with simulated clinical encounters that corresponded directly with concepts under instruction. Students practiced with 12 AI-simulated patients across the quarter, 9 (75%) of which were adapted from Skillsetter transcripts and 3 (25%) adapted from documented examples of sexually inappropriate behaviors toward clinicians reported in prior empirical work [31] (Multimedia Appendix 1). CARE’s scenarios encompassed alliance ruptures, mandated reporting, and crisis management related to suicidal ideation and self-injury, thereby anchoring AI practice in clinically relevant challenges.

Prior to classroom deployment, CARE’s AI-simulated patients underwent an iterative review process conducted by the TA to ensure clinical appropriateness, ethical safeguards, and pedagogical alignment. Each AI-simulated patient was programmed to follow structured behavioral principles, with particular emphasis placed on scenarios involving emotionally sensitive content areas (eg, trauma and self-harm) to verify that AI-generated responses were trauma informed, ethically sound, and supportive of therapeutic learning objectives. Additional realism was introduced through varied voice models designed to convey emotional depth and natural conversational flow. Some identified limitations, including emotional flatness in voice-based responses, were addressed by adding principles to the voice-based AI-simulated patient’s prompt (eg, “Mention past attempts to please family that failed, with resigned sighs, and even breaths where the nose is runny as in crying”).

#### Measurements

#### Surveys

##### First Course

Students completed a midcourse survey in the first course, halfway through the introduction of CARE, and again at the end of the course. The 37-item questionnaire was specifically designed by the investigators for this study to gather insights into the challenges and obstacles students faced while using CARE, as well as their experiences with peer feedback and AI feedback on their peer roleplays. The full set of 37 items for the first course study is provided in Multimedia Appendices 2 and 3.

##### Second Course

To evaluate the comparative value of CARE and Skillsetter, students completed a 39-item investigator-designed survey at the end of week 16 in the second course. Items were adapted from national and professional surveys where possible [36,37]. Students rated their experiences across domains using 7-point Likert scales (1=strongly disagree to 7=strongly agree) and provided open-ended responses. Example items by domain are shown in Table 2. The full set of 39 items for the second course is provided in Multimedia Appendix 4.

**Table 2.** Example survey items from the web-based survey administered in the second course.

Domain	Example items
Comparative value (RQ^a^1)	“The way I could use [CARE/Skillsetter] allowed me to make mistakes I could learn from.” “After conversing with the patients in [CARE/Skillsetter], I feel more equipped to build and maintain a therapeutic alliance.”
AI feedback (RQ1)	“Receiving AI feedback shortly after completing CARE practice enhanced my learning experience.” “I know when to ignore AI feedback when it is incorrect or does not make sense.”
Self-reflection	“I found the self-reflections after a [CARE/Skillsetter] session valuable for understanding how to improve.”
Limitations and design (RQ2)	“My concerns about CARE AI’s patients include: voices sound robotic; transcripts are inaccurate.” “My concerns about CARE AI’s feedback include: wish it tracked progress over time.”
Course integration (RQ3)	“Would you recommend CARE AI to next year’s PsyD cohort as a deliberate practice option?”
Attitudes about AI (RQ4)	“Overall, would you say the increased use of AI in daily life makes you feel… (concerned vs. excited)?” “Do you think AI in the classroom would lead to better or worse helping skills for therapists?”

a RQ: research question.

### Instrument Development

Both surveys were investigator designed for this deployment, as no validated instrument exists for assessing student experiences with AI-augmented DP in psychotherapy training. Items were developed by the research team, which combined expertise in psychotherapy training and in AI systems for clinical education; the instructor and coinvestigators drew on prior experience teaching this course sequence to anticipate likely obstacles, including discomfort with recorded practice, concerns about data use, and skepticism about automated feedback on relational skills. Closed-ended response options for items on limitations and concerns were generated from these anticipated obstacles and from issues students raised during the first course. Items on general AI attitudes were adapted where possible from national and professional surveys [36,37]. The first-course survey served as a formative pilot, and its results informed revision of the second-course instrument.

No formal psychometric evaluation was conducted. Items were not subjected to cognitive interviewing, factor analysis, or reliability testing, and we report each item individually rather than computing scale scores. Quantitative findings should therefore be read as structured summaries of student reports rather than as validated measurements.

### Data Analyses

#### Quantitative Analyses

We conducted nonparametric paired hypothesis tests (2-tailed Wilcoxon signed-rank) to evaluate students’ comparative perceptions of CARE versus the 2 traditional DP modalities: peer role-play feedback and Skillsetter (RQ1). Our investigation concerns different dimensions of student perceptions (eg, comfort in receiving feedback from AI vs peers and immersiveness of voice-based AI and video-based vignette practice) that we consider equally and independently rather than as surrogate indicators of an omnibus finding; therefore, each dimension defines an individual statistical hypothesis tested at an ⍺ of .05 level without correction [38,39]. There were no missing data to account for in these paired survey responses. Statistical testing was conducted using the SPSS package (version 29; IBM Corp). Descriptive statistics are reported for the remaining quantitative items, covering perceived limitations and design needs of CARE (RQ2), perceptions of course integration (RQ3), and attitudes toward AI (RQ4).

#### Qualitative Analyses

Open-ended survey responses were analyzed using reflexive thematic analysis following Braun and Clarke [40,41]. Coding was hybrid: codes were generated inductively from the response data, while the 4 research questions, which structured the survey instrument and therefore the domains in which students responded, oriented the analysis. Two authors (EC and RL) independently read the full response set for familiarization and independently coded all responses. They then met over 3 rounds to compare codes, discuss divergent readings, and develop shared interpretations of the data. Rather than resolving differences by adjudication or by calculating interrater reliability, we treated points of divergence as analytically generative, working through them in discussion until we arrived at interpretations both coders found defensible. This is consistent with the assumptions of reflexive thematic analysis, which positions researcher subjectivity as a resource rather than a source of bias to be controlled [42]. Candidate themes were then developed from the coded material, reviewed against the coded extracts and the full dataset, refined, and named.

#### Classroom Memos

Throughout the deployment, the TA and the co-first author kept informal written notes documenting classroom interactions, student questions raised during class, and technical problems reported in real time. Notes were written within 24 hours of each class session and were not systematically coded or analyzed as a data source. They served 2 limited purposes: to document implementation events that would otherwise go unrecorded (eg, the midquarter decision in course 2 to add supplemental TA feedback in response to student concerns) and to help the analytic team interpret survey responses that referenced specific classroom occurrences. We do not draw findings from the memos and do not treat them as qualitative data; where they inform the narrative, this is stated explicitly in the text.

#### Integration of Quantitative and Qualitative Findings

We used a convergent design in which both strands were collected concurrently within the same surveys and analyzed separately, then integrated at the interpretation stage [43]. Integration proceeded by research question: for each RQ, we compared the direction and strength of the quantitative findings against the corresponding themes, noting points of convergence, divergence, and expansion. Convergence and divergence are reported explicitly in the “Results” section and revisited in the “Discussion” section.

#### Reflexivity

Our team’s composition shaped both the deployment and its interpretation. The team includes computer scientists who developed CARE and its underlying models and clinicians who teach in the training program where the study took place. As developers, we had an investment in CARE functioning well. As instructors and program faculty, we held evaluative authority over the students whose experiences we report. Both students’ willingness to voice criticism and our readiness to hear it were conditioned by these relationships.

Several steps mitigated these risks. Research participation was decoupled from course participation; CARE was made optional in the first course as a supplement to peer feedback on students’ peer role-plays. In the second course, CARE was initially required; however, in the second week of the course, the instructional team made participation with CARE optional due to student concerns, and students could opt to complete weekly DP assignments using the Skillsetter video-based package only.

In coding and interpretation, the 2 coders included 1 coauthor without a role in developing CARE and 1 without. This positioning also bounds what the paper claims. We report students’ perceptions rather than an efficacy evaluation of CARE, and we treat students’ reluctance and ethical objections as findings in their own right rather than as barriers to be overcome.

## Results

### Overview

This section outlines the findings from the 16-week deployment, structured to provide a clear progression of insights. We begin with the first course 2024 pilot study, which served as a preliminary investigation into students’ perceived value of AI feedback as a supplement to peer feedback. We then move to the more extensive second course study, which builds upon the first course to explore AI-simulated patients in greater depth. This ordering reflects the evolving nature of the research and the increasing complexity of the questions addressed.

### First Course 2024 Pilot: AI Versus Peer Feedback

The first course was a formative phase of the study in which we sought to understand students’ experiences of AI-generated feedback and traditional peer feedback following peer role-play exercises. Of the 29 enrolled students, 23 uploaded at least one peer role-play transcript to CARE and 22 received AI feedback on at least one transcript (Table 3). All findings reported below reflect survey respondents only. The survey was voluntary in this course, and 14 (48%) students completed it. The following results are based on 14 survey respondents, 5 of whom did not use CARE by choice or due to technical issues, leaving 9 students who reported on the experience of AI versus traditional peer feedback.

**Table 3.** Prespecified implementation (feasibility) indicators by course*.*

Indicator	Definition	Course 1 (CARE optional)	Course 2 (CARE mandatory in the first week)
Adoption	Percentage of enrolled who used CARE ≥1 time	22/29 (76%) received CARE feedback on their peer role-play	25/29 (86%) used CARE’s AI-simulated patient (mandatory)
Reach	Percentage of enrolled providing any research data	14/29 (48%; survey 1); 9/29 (31%; survey 2)	25/29 (86%)
Completion rate^a^	Percentage of possible assignments completed with CARE with ≥4 dialogue exchanges	73/174 (42%)	304/319 (95%)
AI feedback review rate	Percentage of eligible transcripts that received AI feedback	63/73 (86%)	248/304 (82%)
Completion per student, mean (SD)	Sessions/uploads per student across the course	3.17 (2.15)	10.13 (4.69)
AI feedback reviews per student, mean (SD)	Sessions with feedback per student across the course	2.74 (2.14)	8.27 (3.91)
Assignment substitution	Whether CARE replaced or supplemented existing work	Optional add-on to peer role-play (no substitution)	Additional Skillsetter
Technical transcription failures	Rate of failed/unusable sessions due to transcription errors, including transcription to non-Latin scripts	Logs: non-Latin script in transcription, 0; transcript repetitions, 2 Survey: 1 user reported incorrect diarization	Logs: non-Latin script in transcription, 4; missing speaker, 93; AI patient stopped replying, 44 (midconversation, 15; start of conversation, 29) Survey: Voice-pause detection, 20/25 (80%) users; transcript inaccuracy, 10/25 (40%) users
Time required	Setup and per-session time for students/staff	10 minutes	10 minutes
TA workload	Staff hours for setup, scenario review, and feedback	Scenario review and troubleshooting: 1‐2 hours per week	Weekly written feedback to 3‐5 students and review: 1‐2 hours per week

a Course 1 completion rate assumes 6 chances to upload weekly peer role-plays to CARE×29 participating students; the rate is deflated because CARE use was optional and combines high-usage and no-usage students. Course 2 completion rate assumes 319 required assignments (11 required assignments×29 participating students); the rate is inflated because some students completed multiple sessions per week.

#### RQ1: AI Feedback Versus Peer Role-Play

Quantitative comparisons from the first course pilot (Table 4) show that students rated peer feedback significantly higher than AI feedback along 2 of the 3 dimensions. Specifically, peer partners’ feedback was rated as being more constructive and helpful to a great extent versus a moderate extent (median 3, IQR 3-3 vs median 2, IQR 2-2 on a 0‐4 scale; *P*=.02). Students’ peer partner feedback allowed them to better recognize areas to improve versus CARE with Likert ratings differing between moderately agree and slightly agree (median 5, IQR 5-6 vs median 4, IQR 4-5; *P*=.046). No statistically significant difference was found with regard to their comfort receiving feedback; the median student felt comfortable receiving feedback from peers and CARE’s AI system (median 6, IQR 6-6 vs median 6, IQR 5-6 on a 1‐6 scale). Nonetheless, peer partner feedback had higher mean and lower variance than CARE in terms of comfort (mean 5.88, SD 0.33 vs mean 5.44, SD 0.73), which indicates that a subset of students felt less comfortable with AI feedback.

**Table 4.** Comparing peer role-play practice followed by AI feedback (CARE) versus peer feedback (peer partner) rated by students in the first course (n=9).

Question and modality	Mean (SD)	Median (IQR)	*P* value^a^
Constructive and helpful^b^			.02
CARE	2 (0.707)	2 (2-2)	
Peer partner	3 (0.707)	3 (3-3)	
Comfort receiving feedback^c^			.10
CARE	5.444 (0.726)	6 (5-6)	
Peer partner	5.889 (0.333)	6 (6-6)	
Recognize areas to improve^c^			.046
CARE	4.333 (0.866)	4 (4-5)	
Peer partner	5.222 (0.833)	5 (5-6)	

a *P* values are from Wilcoxon signed-rank tests comparing CARE and peer partner responses.

b Rated on a 0-4 scale (0=not at all, 1=to a small extent, 2=to a moderate extent, 3=to a great extent, and 4=to a very great extent).

c Rated on a 1-6 forced Likert scale (1=disagree strongly, 2=disagree moderately, 3=disagree slightly, 4=agree slightly, 5=agree moderately, and 6=agree strongly).

Qualitatively, students valued peer feedback for its ability to capture nonverbal cues and provide a patient-centered perspective. One student noted, “I think peer feedback considers the whole session, including tone, nonverbals, use of silence, etc, so it feels more relevant as a whole.”

#### RQ2: Design Limitations

Students identified both technical and substantive limitations in CARE’s design. Transcription errors, often linked to audio quality, sometimes undermined the utility of feedback: “There are occasional issues with the transcript... CARE is unable to identify or differentiate between patient and patient at times, resulting in occasionally inaccurate feedback.” Beyond technical issues, participants described the scope of feedback as narrow, with repeated emphasis on empathy and validation while overlooking other therapeutic strategies. As one noted, “Most of the feedback was suggesting that I empathize with and validate the patient, but there are many other techniques... which the AI doesn’t seem to recognize.” Others emphasized that responses were evaluated in isolation: “It feels like each response is being considered separate and apart from the rest, so it feels less relevant to the content overall.”

Despite these concerns, students highlighted the value of alternative phrasing and immediate suggestions. One remarked, “I like the alternative responses... it gives me ideas for how to better and more concisely word my responses.” Another added, “I appreciate how content-oriented AI is! I find the alternative responses extremely helpful as I can use certain phrases in future sessions.” While some described the system as “robotic” or “less personal,” others appreciated its consistency, contrasting it with the subjectivity of human feedback: “Human feedback might only apply for that individual client... I think AI gives broader and more objective feedback that applies to the situation instead of the person.”

#### RQ3: Course Integration

The integration of CARE into the curriculum was shaped by instructional stance and student perceptions of the research team. In the first course, CARE was introduced under exploratory conditions, which encouraged discussion. Use of CARE’s role-play transcript feedback was optional, and its role was described as exploratory. Of the 29 students enrolled in the first course, 14 (48%) completed surveys about the integration of CARE into the course, and 9 (31%) provided comparative ratings about peer roleplay versus CARE.

When asked what prevented them from using CARE during the course, students most frequently cited concerns about privacy and data security, a preference for receiving feedback from a human rather than AI, uncertainty or lack of guidance on how to use the tool, and apprehension about the nature of the research being conducted by the research team (eg, “making a chatbot therapist”).

#### RQ4: Acceptance

A key concern raised by students in the first course was the security and intended use of their video transcripts when uploaded to the CARE platform. To address these concerns, the study team provided detailed explanations during class and in the frequently asked questions (FAQs) document (Multimedia Appendix 5). Students were informed that all video transcripts would be stored on a secure, password-protected cloud server. In rare cases where transcription errors occurred, a member of the research team would access the server to identify and troubleshoot the affected audio file. Aside from this limited quality control function, students were assured their audio files would not be used for any other purpose and would be deleted upon the completion of research analyses. Similarly, text transcripts were to be stored on a secure, password-protected server within CARE’s internal database. These transcripts were accessible only to the student and their selected peer partner. If students opted to use the “Review Feedback” tool, their transcripts were processed by CARE’s AI feedback model. Importantly, CARE’s model is entirely self-hosted and does not rely on commercial AI services (eg, ChatGPT), ensuring that student data are not used to train external proprietary models.

In addition to these measures, students were provided with a formal study information sheet outlining all data protections and ethical commitments associated with the project.

### Second Course: DP

#### Overview

Building on the first course, the second course study shifted focus to the use of CARE’s voice-based AI-simulated patients for DP, comparing this to Skillsetter’s video-based practice. CARE enabled dynamic, back-and-forth dialogue with AI-simulated patients, whereas Skillsetter required a single, one-time response to a video prompt.

Students had already been introduced to the CARE platform in the prior term. The difference for the second course was that students could use CARE for DP, speaking with AI-simulated patients. Students could also receive AI feedback on the basic counseling skills used in their interactions with the AI-simulated patients. However, the focus was on the utility of the DP itself rather than the feedback.

#### RQ1: Comparative Value of CARE Voice-Based AI-Simulated Patients and Skillsetter Video Vignettes

As presented in Table 5, the only significant difference between modalities was learning from mistakes, which students rated higher for Skillsetter than CARE (mean 4.52, SD 2.00 vs mean 3.20, SD 2.04; *P*=.03; *d*=0.49). Descriptively, the remaining comparisons diverged in direction rather than favoring one modality overall: Skillsetter was rated slightly higher for immersion (mean 4.16, SD 1.97 vs mean 3.88, SD 2.05) and confidence responding spontaneously (mean 4.52, SD 2.10 vs mean 4.32, SD 1.82), whereas CARE was rated slightly higher for communication beyond words (mean 4.20, SD 1.96 vs mean 3.80, SD 2.02) and building a therapeutic alliance (mean 4.52, SD 1.85 vs mean 3.80, SD 2.26). These differences were small relative to the variability in responses (SDs ≈ 1.8‐2.3), and none approached significance. Qualitatively, students reported that CARE’s AI-simulated patients provided a “good opportunity to practice using the skills learned in class” and allowed for interactive dialogue, which is not possible with Skillsetter. One student noted they used the AI-simulated patients because “you don’t get to explore the back and forth with Skillsetter.”

**Table 5.** Perceived value of challenging patient scenario practice with traditional video vignettes and instructor feedback (Skillsetter) compared to voice-based AI-simulated patients and AI feedback (CARE) rated by students in the second course (N=25).

Question and modality	Mean (SD)	Median (IQR)	*P* value^a^
Learning from mistakes^b^			.03
CARE	3.2 (2.041)	3 (1-5)	
Skillsetter	4.52 (2.002)	5 (3-6)	
Immersion in interaction^b^			.66
CARE	3.88 (2.048)	4 (2-5)	
Skillsetter	4.16 (1.972)	4 (2-6)	
Communication beyond words^b^			.58
CARE	4.2 (1.958)	4 (3-6)	
Skillsetter	3.8 (2.021)	4 (2-5)	
Confidence responding spontaneously^b^			.80
CARE	4.32 (1.819)	4 (3-6)	
Skillsetter	4.52 (2.104)	5 (2-7)	
Building a therapeutic alliance^b^			.13
CARE	4.52 (1.851)	5 (3-6)	
Skillsetter	3.8 (2.255)	4 (2-6)	

a *P* values come from Wilcoxon signed-rank tests comparing CARE and Skillsetter modalities.

b Rated on a 1-7 scale (1=strongly disagree, 2=disagree, 3=somewhat disagree, 4=neither agree nor disagree, 5=somewhat agree, 6=agree, and 7=strongly agree).

While the second course did not include a peer role-play component, some students reflected on peer roleplay in the open-ended survey responses in course 2. CARE was also valued for simulating difficult scenarios that would be hard to practice with peers, such as dealing with an “angry client, silent client.” Despite these benefits, a strong preference for practicing with humans remained. Students felt that AI practice could not replicate the nuances of human interaction, with one stating, “Nothing replaces role plays.” There were also concerns that skills developed with the AI might not transfer to real therapy, with one student stating they had “learned how to orient towards receiving positive feedback from CARE rather than toward providing thoughtful and nuanced support for a human person.”

#### RQ2: Design Limitations

The most prevalent technical concern (20/25, 80% of respondents) was that the system does not detect vocal pauses, indicating a significant gap in the speech recognition capabilities. More than half of respondents (14/25, 56%) felt that the AI-simulated patients did not feel realistic, with a similar number (13/25, 52%) noting limited emotional expression in the voices. Transcript inaccuracy (10/25, 40%), robotic-sounding voices (9/25, 36%), lack of visual avatar representation (7/25, 28%), and voice recognition issues (6/25, 24%) were additional functionality concerns raised by participants.

Students expressed consistent concerns about the quality and personalization of AI feedback. The 3 top concerns received the same high level of response (17/25, 68%): students wished the AI-simulated patient could provide feedback from its perspective on how the student, as a therapist, made them feel. Students desired a system that tracked and referenced their progression over time. Many reported that the AI was providing unhelpful feedback. Nearly two-thirds (16/25, 64%) of respondents wished the feedback addressed the session as a whole. There was a desire for greater variety in evaluation criteria (15/25, 60%) and feedback that incorporates vocal elements rather than just written transcripts (13/25, 52%).

In response to students’ concerns about the limited utility of CARE’s feedback, the teaching team implemented a supplemental feedback process in which the TA provided individualized written feedback on students’ CARE transcripts. Each week, 3 to 5 students were randomly selected to receive this feedback, which was designed to enhance learning by integrating human insight with AI-generated suggestions. The TA provided weekly written feedback to each student who elected to use CARE, then emailed students individually to ask permission to post their feedback, identified by name, on the course Canvas (Instructure) page. Consenting students’ feedback was posted to a discussion thread so that all students could view it, contribute to the discussion, and potentially benefit from it. The decision to supplement CARE in this way was informed by feedback indicating that the AI’s responses alone were often perceived as insufficient for skill development. This feedback was obtained and documented in classroom memos by the TA.

One student reflected on the benefits of this hybrid model:

*Additionally, I think everything involved with AI would have a better outcome when there is that human aspect. [TA] ended up providing additional feedback for us weekly for CARE. This combined the AI aspect with the human aspect, so it wasn’t only AI that we were interacting with. I feel like this maximized our learning. It would also have been interesting if we actually looked at some of the AI feedback and analyzed those using a forum, in addition to human feedback. I think doing so would again emphasize the aspect of human and AI [or AI-assisted human feedback], instead of just AI doing all the work. AI’s benefits wouldn’t come alive if there weren’t human input to modulate it.*

This perspective underscores students’ desire for feedback that reflects both the analytical consistency of AI and the nuance of human clinical judgment.

#### RQ3: Factors About Course Integration That Impacted Acceptability

The strongest concerns about CARE’s integration into the classroom centered around how AI might replace human elements of education (Table 6), with 22 (88%) of 25 respondents worried about AI replacing peer support and feedback, and 20 (80%) of 25 respondents concerned about AI replacing peer interaction. Many students (19/25, 76%) questioned whether they were simply user-testing a product rather than engaging in an educational experience. Students raised concerns about potential environmental impact (16/25, 64%), suggesting awareness of the computational resources required for AI systems, including potential environmental impacts.

**Table 6.** Concerns about how CARE was integrated into the classroom experience.

Concern	Choice count (N=25), n (%)
AI replacing peer support and feedback	22 (88)^a^
AI replacing peer interaction	20 (80)^a^
Am I just doing user testing for a product	19 (76)^a^
Environmental impact	16 (64)^a^
How my data is used by the research team	14 (56)^a^
What if they are making an AI therapist to replace me	12 (48)
I feel that my data is going to be used to make an AI therapist	12 (48)
Other concerns	7 (28)

a Values indicate fields where more than half of the 25 respondents indicated concerns, with students able to select multiple concerns.

Data usage concerns were prominent, with 14 (56%) of 25 respondents worried about how their data are being used by the research team, and 12 (48%) of 25 respondents concerned about their data potentially being used to train AI therapists.

#### RQ4: Acceptance

##### Overview

In addition to items about CARE, the second-course survey asked about AI in psychotherapy education and clinical practice generally. The responses below address this broader domain and should not be read as evaluations of CARE. Students articulated a range of perspectives, coalescing around three thematic domains: (1) openness to AI integration in training and administrative contexts, (2) boundary concerns regarding the application of AI in clinical care, and (3) broader ethical apprehensions related to AI’s societal impact.

Several students expressed cautious optimism about the integration of AI into psychotherapy, particularly in training contexts and administrative functions. For these individuals, AI was not viewed as a threat to the therapeutic profession, but rather as a supplemental tool that could enhance learning and reduce burdens associated with nonclinical tasks. As one student noted, “I do not personally hold the fear of AI replacing therapists, so the training and administrative help resonates with me.” Another student highlighted the relevance of technological adaptability in a rapidly evolving professional landscape:

*As trainees in therapy, I truly see the importance of adapting to these changes and evolving with our society at large. Eventually, there will be other therapists who utilize AI, and we will be competing with those who know how to utilize it.*

##### Openness to AI Integration in Training and Administrative Contexts

Administrative utility was a recurring subtheme, with several students noting the promise of AI in alleviating logistical tasks such as billing, insurance coordination, and website maintenance. One student proposed that “a better focus for AI in psychotherapy would be having an AI handle the billing, insurance, outreach, and website management that is non-billable time for therapists, rather than training.” Others concurred, suggesting that AI could support clinical infrastructure without compromising the therapeutic relationship.

##### Boundary Concerns Regarding the Application of AI in Clinical Care

Despite general receptivity to AI in administrative and educational settings, many students expressed strong reservations about potential clinical applications affecting direct patient care. A recurrent concern was that incorporating AI into training contexts might normalize its use in psychotherapy delivery, thereby facilitating a negative chain of future events. One student remarked, “Honestly, [I am] a bit more concerned, as I think while the benefits are there, it is a slippery slope.” Another reflected, “I still feel uncertain about... the possibility of AI eclipsing in-person therapy in the future.”

This apprehension was often anchored in the perceived irreplaceability of human relational dynamics. As one student articulated, “Nothing can replace in-person practice and human interaction. Beyond certain admin tasks, I do not think AI should be used in clinical practice.” Another student emphasized the importance of ethical containment: “It’s still important to be cautious with it, and it should never replace an actual human in any context other than training.” These sentiments underscore a shared appreciation for the therapeutic alliance and a desire to establish clear boundaries for emerging technologies.

##### Broader Ethical Apprehensions Related to AI’s Societal Impact

A third thematic domain reflected broader ethical considerations about AI’s proliferation across societal and global systems. For some students, skepticism about AI in psychotherapy was rooted not solely in clinical concerns but also in a larger critique of AI’s sociopolitical implications. One student remarked, “It has increased my concerns about the increase of AI use throughout the world.” Another described their initial discomfort with the study’s AI component, stating, “As someone who has bigger-picture ethical concerns about AI, in relation to environment, jobs, and broader social/societal impact, I was initially pretty upset about having CARE be a required part of this course.”

Concerns about commercialization, technological determinism, and the disconnect between developers and end users were also prominent. One student stated, “The lack of awareness about therapy for those who are developing models frankly terrifies me,” while another wrote, “I feel very hesitant about the use of AI in any space in psychotherapy. To me, it feels like too slippery of a slope, and from history, it seems like things like this inevitably end up in the wrong hands, even if that is not the starting intention.” Reflecting these reservations, some students reported electing not to use the AI platform for training, citing “broader ethical concerns around AI and its rapid expansion.”

Together, these themes reflect a complex and multidimensional stance among emerging psychotherapists. While students acknowledged the potential utility of AI in specific educational and administrative contexts, they also articulated clear boundaries for its use in clinical care and raised critical ethical questions about its integration into psychotherapy and society more broadly.

## Discussion

### Principal Findings

This study examined how doctoral psychotherapy students experienced AI-augmented DP embedded across 2 required courses in a first-year psychotherapy sequence at an APA-accredited clinical psychology program. The 2 courses involved different configurations. The first course served as a formative pilot in which students completed weekly peer role-plays, and CARE was introduced only as an optional source of AI-generated feedback on the resulting role-play transcripts, supplementing the feedback students already received from their peer partners. The second course introduced AI-simulated patients as a practice modality and placed 2 bundled packages side by side, matched for weekly curricular objectives: CARE, comprising multiturn spoken dialogue with voice-based LLM-driven AI-simulated patients followed by automated feedback, and Skillsetter, comprising single-turn recorded responses to standardized video vignettes performed by actors, with instructor and TA feedback.

Three findings stand out across the sequence. First, uptake appeared to be governed less by platform performance than by students’ prior dispositions toward AI. In the first course pilot, 23 (79%) of 29 students uploaded at least one peer role-play transcript, but privacy concerns and a preference for human feedback were the reasons most frequently cited by those who did not, and comparable apprehension led the instructional team to adjust the second course’s initial requirement after the first week, so that students could elect to complete additional Skillsetter practice sessions instead of CARE. Second, students’ comparative ratings of the 2 second-course packages converged on most dimensions, with only learning from mistakes differing significantly in Skillsetter’s favor, while their open-ended responses expressed a clear and consistent preference for practicing with humans. Third, the concerns students raised were largely not instructional; the most frequently endorsed were that AI would displace peer support and peer interaction. Taken together, these findings suggest that the principal obstacles to embedding AI-augmented practice in this curriculum were relational and ethical rather than technical, although technical limitations were substantial and may have compounded them.

### Insights From the First Course Pilot

Students rated peer feedback significantly above CARE’s AI feedback on being constructive and helpful, and on recognizing areas to improve, attributing peers’ advantage to the capacity to integrate tone, nonverbal information, and a patient-centered stance. As CARE operated on transcripts, much of what students valued in peer feedback was information the system could not access, a constraint shared by LLM-based feedback systems relying on textual representations of therapy [12,22,44]. The comparison may therefore have been less between human and machine judgment than between judgment informed by the full session and judgment informed by a degraded record of it.

Students’ explanations nonetheless resemble 2 documented patterns. Research on algorithm aversion finds that people lose confidence in algorithms more rapidly than in people after comparable errors [45], which may help explain why transcription failures appeared to discredit CARE’s feedback more broadly than those specific errors would warrant. Longoni et al [46] report that resistance to medical AI is associated with uniqueness neglect, the concern that automated systems cannot accommodate individual circumstances; our students described AI feedback as evaluating responses “separate and apart from the rest,” while peer feedback “considers the whole session.” We draw on these literatures interpretively because our design tested neither mechanism. Notably, 1 student inverted the framing entirely, valuing AI for giving “broader and more objective feedback that applies to the situation instead of the person,” a divergence cautioning against treating preference for human feedback as uniform.

### Comparative Value of AI-Simulated Patients and Video Vignettes

Students’ ratings of the 2 packages appeared to converge on most dimensions. Skillsetter was rated significantly higher only for learning from mistakes, while immersion, confidence responding spontaneously, communication beyond words, and building a therapeutic alliance did not differ significantly, the latter 2 showing numerically higher means for CARE. Given wide variability and limited power, these patterns are best read as descriptive.

Students also reported concerns about realism, with 56% (14/25) indicating that the AI-simulated patients did not feel realistic, and 52% noting limited vocal expression. Simulation scholarship suggests one possible reading, having generally found fidelity to be largely independent of educational effectiveness, and proposed that functional task alignment may explain outcomes better than physical resemblance [47], with work in mental health simulation reporting psychological fidelity as more critical to learning than physical fidelity [48]. Students may perhaps have experienced the simulated patients as unconvincing depictions of people, while still finding the task aligned with what the course asked them to rehearse.

A notable feature of the open-ended responses was that students introduced a comparison the survey did not ask about. The second-course items concerned CARE and Skillsetter only, and peer role-play was not a component of that course, yet several students referenced it unprompted, with 1 respondent stating that “nothing replaces role plays.” One interpretation is that live practice with a person functioned as an implicit reference point against which both packages were evaluated, although neither offered it. This would be broadly consistent with reports from other relational health professions, where trainees describe AI as acceptable in a supportive role but maintain that it cannot substitute for human-centered interaction and therapeutic communication [49,50]. Some students additionally expressed concern that practicing with AI might lead them to optimize for system-specific feedback rather than responsiveness to a person, an apprehension that appears to parallel deskilling concerns noted in health professions education [51]. Ratings and written responses thus seemed to diverge, with the latter suggesting a preference that the comparative items may not have been positioned to capture.

### Identity-Based Rather Than Integrity-Based Resistance

The concerns our students raised differ in kind from those documented among students in general higher education, where reservations about generative AI cluster around accuracy, academic integrity, and overreliance diminishing critical thinking [52]. These were largely absent from our data. No student framed CARE as a shortcut around effort or worried that using it would undermine their own learning. Their most endorsed concerns were that AI would replace peer support and feedback (22/25, 88%) and peer interaction (20/25, 80%), and nearly half worried their data might build an AI therapist that could replace them. The object of concern appears to have been not their academic conduct but their professional future.

This pattern resembles reports from other relational health professions more than from general education. A systematic review of nursing students and practicing nurses found consistent argument that AI should augment rather than replace human empathy and judgment, with fear of deskilling impeding adoption [49]; qualitative studies describe AI accepted as supportive but incapable of replacing therapeutic communication [50]. Jussupow et al [53] offer a possible mechanism, reporting that threats to professional recognition and to professional capabilities predicted resistance among medical students and physicians, with students experiencing stronger identity threats than experienced clinicians. Students whose developing expertise is defined by relational work may appraise AI in that domain partly as a threat to the identity they are acquiring rather than solely as a tool of uncertain utility. If so, this resistance may call for different responses than those developed for general education. Guidance on academic integrity and appropriate reliance addresses concerns our students did not appear to hold; what they voiced was uncertainty about whether the relational core of their profession would be preserved. Such concerns seem more tractable through transparency about a system’s purposes, framing that positions AI as supplementing rather than substituting for human relationship, and protection of the peer and supervisory interactions students valued most. We note the limits of this comparison: we sampled 1 cohort in 1 program and rest the contrast on triangulation against published work rather than on data of our own.

### Design Limitations and Opportunities

Students’ design concerns appeared to center on feedback that did not attend to the session as a whole. Three were endorsed equally often (17/25, 68% each): that the simulated patient could not report how the student’s responses made it feel, that progress was not tracked over time, and that feedback was frequently unhelpful; 64% (n=16) wanted feedback addressing the whole session. Prior work suggests that pairing simulated practice with feedback is both desired [23,26] and associated with skill improvement [21,22]; sustained multiweek use may additionally surface a need for criteria matched to each week’s pedagogical goal rather than to a fixed set of listening microskills.

Across both courses, students seemed to prefer not better AI feedback but AI feedback accompanied by human feedback. Peer feedback was rated above AI feedback in the first course, and supplemental TA feedback on CARE transcripts appeared to be received positively in the second, with 1 student observing that “AI’s benefits wouldn’t come alive if there weren’t human input to modulate it.” Hybrid approaches have been examined in general and online education [54,55] and in mental health support [17-19]. As we did not measure skill, our findings cannot speak to whether hybrid feedback improves acquisition, but they may suggest that automated feedback alone was not readily accepted as a substitute in this setting.

### Course Integration

Instructional stance played a critical role in shaping students’ engagement. Concerns about data security and transcript use were salient, leading the team to develop a detailed FAQ and formal information sheet to clarify protections. These lessons guided adjustments in the second course, where CARE was more fully embedded as a core pedagogical component. Incorporating CARE as a weekly course requirement corresponded to higher participation and normalization of its use. However, after the second week of the second course, CARE became optional due to students’ apprehension. They could choose to use CARE for DP or complete additional Skillsetter videos.

This phenomenon aligns with other recent course-based integrations of AI-simulated patients that vary in instructional requirements. Prior work has documented both optional and required approaches to AI-supported practice, with optional implementations associated with more limited uptake and required implementations designed to ensure broad exposure across learners [25,26]. Taken together, these approaches suggest that optional integration may limit reach, whereas required engagement may increase participation and normalize use. At the same time, the present findings indicate that when AI-based practice is required, sustained engagement may depend on parallel instructional investment, including responsiveness to student concerns and the provision of human feedback to contextualize and support AI-augmented learning.

### Acceptance and Broader Perspectives

In addressing acceptance of AI in psychotherapy education (RQ4), students articulated views that were neither uniformly negative nor organized around instructional considerations alone. Several expressed cautious openness, particularly toward training and administrative applications, with 1 proposing that AI might more appropriately handle billing and insurance coordination than training itself. This ambivalence appears broadly consistent with reports that health professions students often hold simultaneously positive and apprehensive attitudes toward AI [56], and with findings in nursing that trainees accept AI as a supportive tool while resisting it in more central roles [49,50]. It also cautions against characterizing this cohort as uniformly resistant.

Where students did express reservations, these seemed to concentrate at the boundary between training and clinical care rather than within training itself. A recurrent concern was that normalizing AI in coursework might facilitate its later migration into practice, described by 1 student as “a slippery slope,” and such apprehension was typically anchored in the perceived irreplaceability of human relational processes widely regarded as foundational to psychotherapy [1,4,35]. Notably, students drew this line at clinical care rather than at learning: their objection appeared to be less that AI cannot support skill development than that its presence in training might legitimize its presence elsewhere. This may distinguish their concerns from those documented in general higher education, where reservations more often center on integrity and overreliance affecting students’ own learning [52].

A third set of concerns extended well beyond psychotherapy and was not anticipated in our design. Students raised commercialization and the perceived disconnect between developers and practitioners, with one writing that “the lack of awareness about therapy for those who are developing models frankly terrifies me.” Environmental impact was endorsed by 64% (n=16) of respondents, a theme that appears to have received limited attention in the psychotherapy training literature to date, and some students reported declining to use the platform partly on these grounds. A few spontaneously referenced the January 2025 Southern California wildfires; because this association was not systematically assessed, we note it only as possible context. Taken together, these responses may suggest that acceptance of a particular educational tool is partly conditioned by students’ appraisal of the broader industry producing it, a consideration that instructional design alone may be poorly positioned to address.

### Limitations

Several limitations should be noted. This study was conducted within a single graduate training program, and the experiences of this cohort may reflect the particular pedagogical environment, instructional style, or program culture in which CARE was introduced. The program’s highly selective nature, which admitted 30 students from 644 applicants during the 2024 to 2025 cycle (a 7.1% admit rate), may further limit generalizability to less clinically experienced graduate students. In addition, the sample consisted of first-year graduate students enrolled in a multiyear program characterized by intensive supervision, including regular review of recorded therapy sessions with experienced supervisors. This context likely shaped student perceptions of AI-assisted training and may limit generalizability to settings where trainees have fewer opportunities for expert feedback (eg, peer counselors or paraprofessionals with limited supervision access).

Beyond the characteristics of the program itself, participation varied within the cohort. The first-course survey was optional and completed by 14 (48%) of 29 students; the second-course survey was a required component of the coursework and completed by 25 (86%) of 29. Students who used CARE and completed the first-course survey may differ systematically from those who did not, particularly given the privacy and data security concerns reported by students who declined the tool, so the 9 students providing comparative ratings of AI and peer feedback are not representative of the cohort. The first course was also shorter, spanning 6 weeks rather than 10, and CARE was an optional supplement rather than an assigned activity. We report these findings as preliminary and include them to provide context for the second-course deployment. Throughout, we describe findings as those of survey respondents rather than of the cohort.

The CARE platform integrated existing technologies for voice-based AI-simulated patients and feedback generation that were best performing in December 2024. Technical issues may have reflected the specific version deployed rather than inherent limitations of AI-assisted training tools: voice-based practice interactions had issues with transcription accuracy, speech recognition, and limited emotional expression, while feedback was limited to skill-based content on patient-centered listening skills rather than scenario-specific guidance regarding therapeutic-alliance challenges.

Our safety procedures relied on the courses’ existing supervisory structures rather than on study-specific monitoring. This design has a defensible rationale: the topics were already part of the curriculum, taught by the same faculty, and practiced concurrently in an established platform, but it means we cannot report a systematic account of whether the AI-simulated patients generated inappropriate content or whether students experienced distress that went unvoiced. Deployments at larger scale, or in settings without an embedded clinical instructor, would require monitoring procedures we did not implement here: routine transcript review, automated flagging of high-risk content, or structured post-session check-ins.

A central constraint on interpretation is that CARE and Skillsetter differed simultaneously along several dimensions rather than a single manipulated variable. The 2 packages contrasted voice-based with video-based interaction, multiturn dialogue with single-turn response, and a research prototype with an established commercial platform. Because these attributes were bundled, the present design cannot isolate the educational contribution of any one of them, including AI per se, voice interaction, dialogic format, feedback source, or platform maturity. We therefore frame our findings as an evaluation of students’ perceptions of 2 bundled instructional packages as actually delivered in the classroom, rather than as a test of “AI versus traditional training.” The comparative ratings should be read as package-level judgments; disentangling the individual active ingredients will require factorial or component-controlled designs in future work.

All outcomes reported here are student self-report. We did not assess objective change in psychotherapy competence, skill acquisition, alliance-building ability, or clinical readiness, and we make no claims that CARE improved these. Perception items (eg, feeling “more equipped to build a therapeutic alliance”) index students’ subjective experience, not measured skill.

### Future Work

The findings and limitations of this study suggest several priorities for future research on AI-assisted psychotherapy training. Short-term work should focus on improving the technical functionality of AI platforms. Students in this study consistently identified transcription errors as well as AI-simulated patients with limited vocal expressiveness as barriers to engagement. Research is needed to evaluate whether improvements in speech recognition, naturalistic prosody, and affective responsiveness increase the realism and pedagogical value of AI-simulated patients.

Parallel efforts should advance feedback systems toward greater context awareness. Current utterance-level approaches offer timely guidance on specific communication skills, but models that process turns independently can produce feedback that is repetitive, contradictory, or disconnected from the broader therapeutic process. Prior work on automated feedback and performance assessment in psychotherapy and counseling has similarly noted that utterance-level or narrowly scoped feedback may be insufficient for supporting integration of relational and process-oriented skills across the flow of a session [12,19,20]. Approaches that consider the entire session history and enforce global consistency during feedback generation could better support students in developing coherent, session-oriented improvements. Moreover, consistent with emerging evidence that human–AI collaborative feedback may enhance contextual understanding and learner trust [17-19], short-term research should further evaluate hybrid feedback models that combine AI-generated suggestions with human commentary, an approach that students in the present study viewed as particularly beneficial.

Beyond technical functionality, future studies should evaluate whether AI-assisted training translates into measurable improvements in clinical skill acquisition. Recent research on AI-supported counseling and psychotherapy training has highlighted the need to move beyond learner self-report toward objective indicators of competence, including supervisor ratings, behavioral coding of microskills, and structured performance assessments [6,21,22]. Critically, such assessments should extend beyond generic skill-based evaluations (eg, patient-centered listening) to include scenario-specific rubrics aligned with the particular competencies being taught, whether therapeutic-alliance navigation, crisis intervention, or other course-specific learning objectives. Experimental designs that directly compare AI-only, human-only, and hybrid feedback approaches would clarify the specific pedagogical contributions of each modality and help determine whether AI-supported practice confers additive benefits beyond established training methods. Multisite studies are also essential to assess whether findings generalize across programs with differing curricula, instructional philosophies, and trainee populations, a limitation frequently noted in early evaluations of AI-based training tools [6,21].

At a broader level, long-term research should address how AI integration reshapes the pedagogy and culture of psychotherapy training. Questions include whether repeated practice with AI-simulated patients influences professional identity formation and sensitivity to relational nuance. Ethical considerations will remain central, particularly regarding transparency in data use, the potential displacement of human elements in training, and environmental sustainability. The salience of ecological concerns in this study, possibly amplified by the January 2025 Southern California wildfires, highlights the need for future work to consider how external sociocultural contexts intersect with classroom-based experiences of AI. Ultimately, research must examine whether AI can be a useful adjunct to psychotherapy training, enhancing current methods, or whether its widespread adoption risks altering the relational foundations of psychotherapy education.

### Recommendations for Classroom Implementation

These recommendations were informed by student feedback across both terms, as well as by the adjustments made between the first course pilot and the second course deployment.

#### Transparency

Clear, proactive communication about data use, research protocols, and pedagogical aims is essential to building student trust. The second course implementation benefited from refinements to communication strategies developed in response to concerns raised during the first course pilot.

#### Hybrid Models

Combining AI feedback with human oversight may mitigate concerns about impersonality and enhance learning outcomes. The addition of supplemental TA feedback during the second course exemplified this adjustment and was positively received by students. Future research may benefit from systematically examining the impact of required AI integration on students’ perceptions of the teaching team, including measures of instructional alliance, trust, and perceived support, to assess whether and how AI-mediated feedback influences the relational dynamics of the learning environment.

#### Instructor Stance

Student reflections suggested that instructional framing influenced their engagement with CARE. In the first course, the tool was positioned as optional following student concerns and apprehension, and several students reported feeling more willing to try it under these conditions than when it was framed as required. In the second course, CARE was initially presented as a central, required component of the course, which corresponded with higher participation early in the term. However, after concerns were raised, the instructional team shifted to making it optional, where students could engage in additional Skillsetter practice instead of CARE. These observations suggest that how the AI tool is introduced and framed by instructors plays a role in shaping student engagement, and that providing choice in an assigned activity can foster greater willingness to engage with AI-assisted training.

### Conclusions

The integration of CARE across 2 academic terms illustrates both the promise and challenges of AI-assisted psychotherapy training. The first course pilot surfaced key concerns regarding feedback quality, course integration, and data transparency, while the second course study demonstrated how iterative adjustments, such as clearer communication, hybrid feedback models, and shifts in instructional framing, enabled more effective implementation. At the same time, students expressed persistent preferences for human-based training and raised concerns about realism, data use, and broader societal implications, including environmental impact. Taken together, these findings suggest that at this stage of development, AI has the potential to serve as a valuable adjunct to psychotherapy education when framed as a supplement to human interaction, implemented transparently, and paired with human oversight.

Looking ahead, priorities for advancing this field can be organized across 3 levels. Short-term priorities involve addressing technical limitations and refining feedback mechanisms. Medium-term priorities focus on evaluating whether AI-assisted practice translates into measurable improvements in clinical performance, ideally through controlled, multisite studies that include objective learning outcomes. Long-term priorities include examining how AI integration reshapes the pedagogy and culture of psychotherapy training, professional identity formation, and ethical considerations such as data use, role displacement, and environmental sustainability.

The utility of AI-augmented psychotherapy training may differ across educational contexts. In this graduate program, students receive intensive supervision, including live or taped review of clinical encounters, which may shape how they perceive the added value of AI. In other settings, such as programs with more limited supervisory resources, AI tools may assume a more prominent role in supporting DP and providing structured feedback.

Thus, the pedagogical value of AI may not be uniform but instead contingent on the broader training environment, with its potential relevance varying according to the availability of human supervision and feedback.

By situating AI within authentic classroom contexts and iteratively adapting implementation based on student feedback, this study provides a strong foundation for future inquiry in psychotherapy pedagogy. Its contribution lies not only in documenting students’ perceptions of AI-assisted training but also in demonstrating how responsive piloting can inform best practices for responsibly integrating emerging technologies into psychotherapy education.

## Supplementary material

10.2196/93199 Multimedia Appendix 1 Descriptions of the 12 AI-simulated patients used on the CARE platform in the second course, by week and topic, with each patient’s vignette and target therapeutic skills.

10.2196/93199 Multimedia Appendix 2 First-course midcourse survey: peer feedback and AI feedback. Questionnaire administered midway through the first course. It assessed CARE use and barriers to use, the perceived helpfulness of and comfort with peer and AI feedback, learning from reviewing a partner’s AI feedback, and the influence of AI feedback on peer feedback exchanges. Items used Likert-type and open-ended response formats, and branching logic routed students according to whether they had received AI feedback on CARE.

10.2196/93199 Multimedia Appendix 3 First-course end-of-course survey: peer feedback and AI feedback. Questionnaire administered at the end of the first course. It covers the same domains as the midcourse survey, with items addressing feedback from each of 2 peer partners and ratings of factors that make feedback exchange effective.

10.2196/93199 Multimedia Appendix 4 Second-course end-of-course survey: comparing CARE and Skillsetter. Questionnaire administered at the end of the second course. It assessed general attitudes toward AI, concerns about CARE’s AI patients, feedback, and classroom integration, and paired comparisons of CARE and Skillsetter across learning from mistakes, immersion, communication beyond word choice, spontaneous responding, and alliance building. It also asked about perceptions of AI feedback and experiences with utterance-level self-reflection. Items used 7-point Likert scales, multiple-choice, and open-ended responses.

10.2196/93199 Multimedia Appendix 5 Frequently asked questions (FAQ) document on CARE data use and privacy. Information document provided to students in the first course addressing why no consent form was required, voluntary participation, how to raise questions, audio transcription, transcript storage and data use, how to request exclusion of specific transcripts, and the skills framework underlying CARE's AI feedback.

## Abbreviations

APA: American Psychological Association
AWS: Amazon Web Services
DP: deliberate practice
FAQ: frequently asked question
GPU: graphics processing unit
IRB: institutional review board
LLM: large language model
RQ: research question
TA: teaching assistant
