# Supplementary material for: Classroom Integration of AI-Simulated Patients and Automated Feedback in Doctoral Psychotherapy Training: Longitudinal Mixed Methods Study
Source: Interact J Med Res. 2026 Nov 11;15:e93199. doi: 10.2196/93199 (PMC13754175; doi:10.2196/93199)
Supplement: Supplementary file 1 [file ijmr-v15-e93199-s001.pdf]

Supplementary Material: AI-Simulated Patient Scenarios Deployed in the Second (Winter) Course

Students in the second course practiced with 12 AI-simulated patients on the CARE platform, mapped onto the weekly curricular topics of the course. Nine scenarios were adapted from existing Skillsetter transcripts, and three (Week 8) were adapted from documented cases of sexually inappropriate behavior toward clinicians reported in the published literature. Each scenario was presented to students with a brief background vignette; the descriptions below summarize the behavioral principles guiding each simulated patient and the skills each scenario was designed to practice.

The 12 scenarios described here are those reported in the main text. The CARE scenario library also contained additional practice patients (e.g., scenarios targeting early alliance formation, confidentiality, and resistance/reactance) that were offered as options alongside these scenarios, so that 2–4 AI-simulated patients were available to students each week across the 10-week deployment (see Table 1 in the main text). These additional practice patients are not described individually in this supplement.

Course weeks refer to the second course's syllabus. Course Weeks 3–8 correspond to Weeks 9–14 of the overall study timeline shown in Figure 1.

AI-simulated patients by course week and curricular topic.

Course Week	Curricular Topic	AI-Simulated Patients
Week 3	Therapist Self-Disclosure	Denise, Derek
Week 4	Cultural Humility, Power, and Identity	Fionna, Aisha, Nolan
Week 6	Alliance Ruptures and Repair	Catherine, Sandra
Week 7	Mandated Reporting and Suicide Risk	Katrina, Phoebe
Week 8	Sexual Attraction and Boundary Maintenance	Claire, Adam, Samantha

Week 3: Therapist Self-Disclosure

Denise — Client desires therapist self-disclosure

A mandated client experiencing substance use and homelessness who discusses experiences of being trans and Indigenous and repeatedly having institutions exert control over her life. She becomes uncomfortable with the asymmetry of therapy and says she will not continue sharing unless the therapist shares something personal too.

Practice emphasis: Navigate a direct request for therapist self-disclosure in the context of mistrust, power imbalance, marginalized identities, and mandated treatment.

Derek — Feeling inadequate and stuck in career

A 37-year-old divorced father and middle manager who feels stuck while others seem to be advancing. He is skeptical of therapy and asks the therapist whether they have personally experienced feeling stuck and how they handled it.

Practice emphasis: Respond to pressure for personal disclosure while maintaining the therapeutic focus, especially when the client frames disclosure as evidence that the therapist can offer something more useful than “textbook” advice.

Week 4: Cultural Humility, Power, and Identity

Fionna — Difficulties adapting to North American culture

A 22-year-old student struggling with belonging after adapting to North American culture and then experiencing reverse culture shock during a return to China. She feels regarded as foreign in China but also feels she did not grow up in North America, leaving her with the sense that she has “no home.”

Practice emphasis: Explore cultural identity and belonging without prematurely interpreting her experience or imposing solutions.

Aisha — Muslim-American student with anxiety towards future

A 19-year-old Muslim American student whose Pakistani immigrant parents sacrificed significantly for her education. Although highly successful as a pre-med student, she feels drawn toward teaching and struggles to reconcile this with family expectations and her sense that Allah may be calling her toward work with children.

Practice emphasis: Explore intersecting family, immigrant, religious, and career values without assuming what any of those identities mean to Aisha.

Nolan — African-American man struggling with depression and family substance abuse

A 28-year-old African American man whose mother struggled with substance dependence throughout his childhood. Discovering that his mother and younger brother are now using heroin together intensifies his depression and connects with broader anger and hopelessness about racial violence.

Practice emphasis: Make space for both family trauma and experiences of racism without reducing one to the other or prematurely shifting toward problem solving.

Week 6: Alliance Ruptures and Repair

Catherine — Navigating ruptures in the therapeutic alliance

A reflective client who notices moments of tension, misunderstanding, or perceived judgment between herself and her therapist and wants to discuss them directly.

Practice emphasis: Recognize and repair alliance ruptures through validation, direct discussion of misattunement, exploration of both parties’ contributions, and rebuilding trust rather than avoiding the tension.

Sandra — Alliance rupture and marital issues

Sandra has been in therapy for several weeks for marital problems but questions whether therapy can actually change anything. She feels judged about drinking that she sees as necessary for professional networking, since socializing and drinking with clients are embedded in her work.

Practice emphasis: Manage defensiveness and an alliance rupture, with the possibility of gradual softening when the therapist successfully rebuilds rapport.

Week 7: Mandated Reporting and Suicide Risk

Katrina — Suicidal ideation and depression

A 24-year-old woman with depression who has begun mentioning thoughts such as stepping in front of a bus, taking extra medication, or going to sleep and not waking up. She is described as not actively suicidal but minimizes and downplays these thoughts.

Practice emphasis: Recognize indirect or minimized suicidal ideation, sensitively elicit fuller disclosure, and respond to hesitation and humor without allowing minimization to obscure the concern.

Phoebe — Elderly woman experiencing abuse from son-in-law

A 72-year-old woman whose son-in-law is caring for her while her daughter travels for work. She indirectly reveals being pushed and left alone but hesitates to disclose more because she fears disrupting her daughter’s career.

Practice emphasis: Detect subtle disclosure of possible elder abuse, explore safety while respecting ambivalence, and work with guilt and reluctance around reporting or involving others.

Week 8: Sexual Attraction and Boundary Maintenance

Claire — Mild flirtation in sessions

A 29-year-old professional in therapy for relationship problems who increasingly compliments the therapist and jokingly suggests spending time together outside therapy. She dismisses the behavior as playful and harmless.

Practice emphasis: Identify and address mild boundary-testing while preserving the alliance; she downplays the behavior rather than overtly challenging boundaries.

Adam — Intense eroticized transference

A 34-year-old man with abandonment and attachment difficulties who increasingly describes the therapist as the only person who understands him and expresses romantic feelings and interest in a relationship beyond therapy.

Practice emphasis: Maintain boundaries while addressing intense attachment and eroticized transference; he experiences boundary-setting as rejection and may respond defensively.

Samantha — Countersexualized humor

A 42-year-old woman in therapy for marital problems who repeatedly directs sexual jokes and innuendo toward the therapist and characterizes them as simply her sense of humor.

Practice emphasis: Address recurrent sexualized humor and its impact on the therapeutic relationship without unnecessarily escalating the interaction; she responds to boundary-setting with dismissiveness and mild annoyance.
