# Supplementary material for: Classroom Integration of AI-Simulated Patients and Automated Feedback in Doctoral Psychotherapy Training: Longitudinal Mixed Methods Study
Source: Interact J Med Res. 2026 Nov 11;15:e93199. doi: 10.2196/93199 (PMC13754175; doi:10.2196/93199)
Supplement: Supplementary file 2 [file ijmr-v15-e93199-s002.pdf]

Mid-class Survey: Peer Feedback and AI Feedback

This questionnaire should take approximately 10 minutes to complete. Please reflect on your experiences engaging in peer role-plays, and receiving and giving feedback in the course.

* Indicates required question

CARE usage

1. Have you registered for CARE & received AI feedback on a role-play transcript? *

   Mark only one oval.

   - ( ) Yes *Skip to question 2*
   - ( ) No *Skip to question 3*

Challenges using CARE

2. Have you encountered any obstacles when trying to use CARE?

   Check all that apply.

   - [ ] Website link doesn't load
   - [ ] Technical difficulties with uploading audio transcript
   - [ ] Unsure how to receive AI Feedback as the therapist
   - [ ] AI Feedback doesn't load
   - [ ] Unsure how to view the AI Feedback my partner received when they were the therapist
   - [ ] Other: ______________________

Skip to question 11

Haven't received AI Feedback on CARE

3. What is preventing you from using the tool? *

Check all that apply.

- [ ] Concerned about privacy or data security
- [ ] Unsure how to use the tool
- [ ] Technical difficulties with the tool
- [ ] Too busy to complete registration/upload
- [ ] Prefer feedback from a human rather than an AI
- [ ] Waiting for more information or guidance on using CARE
- [ ] Other: ______________________

Skip to question 4

Peer Feedback Experience

The next questions ask you about the constructive feedback conversations you have had with your partner in the 1st half of the course

Receiving Feedback from Peers

4. To what extent did you find the feedback from your peer partner to be constructive and helpful? *

Mark only one oval.

- ( ) 0 - Not at all
- ( ) 1 - To a small extent
- ( ) 2 - To a moderate extent
- ( ) 3 - To a great extent
- ( ) 4 - To a very great extent

5. I am comfortable receiving feedback from my peer partner. *

Mark only one oval.

- Disagree Strongly
- Disagree Moderately
- Disagree Slightly
- Agree Slightly
- Agree Moderately
- Agree Strongly

6. The peer feedback has helped me recognize specific areas where I can improve. *

Mark only one oval.

- Disagree Strongly
- Disagree Moderately
- Disagree Slightly
- Agree Slightly
- Agree Moderately
- Agree Strongly

7. Please elaborate on your experience receiving feedback from your roleplay partner (e.g., the value of it, comfort in receiving their feedback, helping your learning)

Giving Feedback to Peers

The next questions ask about when you gave feedback to peers in the past two outside-of-class roleplays

8. It was hard to deliver constructive criticism to my peer honestly. *

Mark only one oval.

- 0 - Not at all true
- 1 - Somewhat true
- 2 - Moderately true
- 3 - Very true
- 4 - Completely true

9. Sometimes, my constructive feedback didn't show how I really felt inside. *

Mark only one oval.

- 0 - Not at all true
- 1 - Somewhat true
- 2 - Moderately true
- 3 - Very true
- 4 - Completely true

10. Please elaborate on your experience giving constructive feedback to your peer roleplay partner. *

Reflecting on Feedback from Peers and AI

For the following questions, reflect on the feedback you received as a therapist in the past two outside-of-class roleplay assignments

11. To what extent did you find the feedback from your peer partner to be constructive and helpful? *

Mark only one oval.

- 0 - Not at all
- 1 - To a small extent
- 2 - To a moderate extent
- 3 - To a great extent
- 4 - To a very great extent

12. To what extent did you find the AI feedback to be constructive and helpful? *

Mark only one oval.

- 0 - Not at all
- 1 - To a small extent
- 2 - To a moderate extent
- 3 - To a great extent
- 4 - To a very great extent

13. I am comfortable receiving feedback from my peer partner. *

Mark only one oval.

- Disagree Strongly
- Disagree Moderately
- Disagree Slightly
- Agree Slightly
- Agree Moderately
- Agree Strongly

14. I am comfortable receiving feedback from the AI feedback system. *

Mark only one oval.

- Disagree Strongly
- Disagree Moderately
- Disagree Slightly
- Agree Slightly
- Agree Moderately
- Agree Strongly

15. The peer feedback has helped me recognize specific areas where I can improve. *

Mark only one oval.

- Disagree Strongly
- Disagree Moderately
- Disagree Slightly
- Agree Slightly
- Agree Moderately
- Agree Strongly

16. The AI feedback has helped me recognize specific areas where I can improve. *

Mark only one oval.

- Disagree Strongly
- Disagree Moderately
- Disagree Slightly
- Agree Slightly
- Agree Moderately
- Agree Strongly

17. Please elaborate on your experience receiving feedback from your roleplay partner (e.g., the value of it, comfort in receiving their feedback, helping your learning)

____
____
____
____
____

18. Please elaborate on your experience receiving AI feedback (e.g., the value of it, comfort in receiving it, helping your learning)

____
____
____
____
____

Partner's AI Feedback (2/4)

Think back to the roleplay sessions where you were the client and your partner was the therapist, and they received AI feedback on their therapy skills.

19. How often did you review the AI feedback given on your partner's therapy roleplay? *

Mark only one oval.

- ( ) I never reviewed the AI feedback on their therapy performance.
- ( ) I reviewed the AI feedback for some of the weeks.
- ( ) I reviewed the AI feedback every week.
- ( ) I reviewed the AI feedback multiple times each week.

20. To what extent was the AI feedback your partner received helpful for your own learning? *

Mark only one oval.

- N/A - I did not view the AI feedback for transcripts where my partner was the therapist
- 1 - Not at all
- 2 - To a small extent
- 3 - To a moderate extent
- 4 - To a great extent
- 5 - To a very great extent

21. Reviewing the AI feedback given to my partner led me to adjust my use of therapy skills. *

Mark only one oval.

- N/A - I did not view the AI feedback for transcripts where my partner was the therapist
- Disagree Strongly
- Disagree Moderately
- Disagree Slightly
- Agree Slightly
- Agree Moderately
- Agree Strongly

22. Please elaborate on any notable learning that occurred from reviewing feedback about your partner's therapist roleplay.

Constructive Feedback Conversations (3/4)

The next questions ask you about the constructive feedback conversations you have had with your partner in the 1st half of the course

23. It was hard to deliver constructive criticism to my peer honestly. *

Mark only one oval.

- 0 - Not at all true
- 1 - Somewhat true
- 2 - Moderately true
- 3 - Very true
- 4 - Completely true

24. Sometimes, my constructive feedback didn't show how I really felt inside. *

Mark only one oval.

- 0 - Not at all true
- 1 - Somewhat true
- 2 - Moderately true
- 3 - Very true
- 4 - Completely true

25. Please elaborate on your experience giving constructive feedback to your peer roleplay partner. *

AI Feedback Influence (4/4)

The next questions ask you how AI feedback has influenced the feedback discussions with your partner

26. To what extent has receiving or viewing others receiving AI feedback changed how you deliver feedback to your peers? *

Mark only one oval.

- 1 - Not at all
- 2 - To a small extent
- 3 - To a moderate extent
- 4 - To a great extent
- 5 - To a very great extent

27. How has the availability of AI feedback affected your comfort level in giving constructive criticism to your peers? *

Mark only one oval.

- Much less comfortable
- Somewhat less comfortable
- No change in comfort level
- Somewhat more comfortable
- Much more comfortable

28. Please elaborate whether AI feedback has influenced how you exchange feedback with your partner. *

This content is neither created nor endorsed by Google.

Google Forms
