# Supplementary material for: Classroom Integration of AI-Simulated Patients and Automated Feedback in Doctoral Psychotherapy Training: Longitudinal Mixed Methods Study
Source: Interact J Med Res. 2026 Nov 11;15:e93199. doi: 10.2196/93199 (PMC13754175; doi:10.2196/93199)
Supplement: Supplementary file 3 [file ijmr-v15-e93199-s003.pdf]

End-class Survey: Peer Feedback and AI Feedback

This questionnaire should take approximately 10 minutes to complete. Please reflect on your experiences engaging in peer role-plays, and receiving and giving feedback in the course.

* Indicates required question

CARE usage

1. Have you registered for CARE & received AI feedback on a role-play transcript? *

   Mark only one oval.

   - ◯ Yes *Skip to question 2*
   - ◯ No *Skip to question 3*

Challenges using CARE

2. Have you encountered any obstacles when trying to use CARE?

   Check all that apply.

   - ☐ Website link doesn't load
   - ☐ Difficulties when uploading recordings
   - ☐ Issues with transcript accuracy
   - ☐ Problems accessing feedback
   - ☐ Other: ______________________

Skip to question 17

Haven't received AI Feedback on CARE

3. What has prevented you from using the tool during this course? *

Check all that apply.

- [ ] Concerned about privacy or data security
- [ ] Unsure how to use the tool
- [ ] Technical difficulties with the tool
- [ ] Too busy to complete registration/upload
- [ ] Prefer feedback from a human rather than an AI
- [ ] Waiting for more information or guidance on using CARE
- [ ] My therapy partner feels uncomfortable uploading sessions to CARE
- [ ] Concerns about the nature of the study that the Stanford CS team was conducting
- [ ] Other: ____________________

Skip to question 4

Peer Feedback Experience

The next questions ask you about the constructive feedback conversations you have had both of your peer partners

Receiving Feedback from Peers

4. To what extent did you find the feedback from your *1st peer partner* to be constructive and helpful? *

Mark only one oval.

- ( ) 0 - Not at all
- ( ) 1 - To a small extent
- ( ) 2 - To a moderate extent
- ( ) 3 - To a great extent
- ( ) 4 - To a very great extent

5. To what extent did you find the feedback from your *2nd peer partner* to be constructive and helpful? *

Mark only one oval.

- 0 - Not at all
- 1 - To a small extent
- 2 - To a moderate extent
- 3 - To a great extent
- 4 - To a very great extent

6. I felt comfortable receiving feedback from my *1st peer partner* *

Mark only one oval.

- Disagree Strongly
- Disagree Moderately
- Disagree Slightly
- Agree Slightly
- Agree Moderately
- Agree Strongly

7. I felt comfortable receiving feedback from my *2nd peer partner* *

Mark only one oval.

- Disagree Strongly
- Disagree Moderately
- Disagree Slightly
- Agree Slightly
- Agree Moderately
- Agree Strongly

8. The feedback from my *1st peer partner* helped me recognize specific areas where I can improve *

Mark only one oval.

- Disagree Strongly
- Disagree Moderately
- Disagree Slightly
- Agree Slightly
- Agree Moderately
- Agree Strongly

9. The feedback from my *2nd peer partner* helped me recognize specific areas where I can improve *

Mark only one oval.

- Disagree Strongly
- Disagree Moderately
- Disagree Slightly
- Agree Slightly
- Agree Moderately
- Agree Strongly

10. Please elaborate on your experience receiving feedback from your role play partners (e.g., how it compared between partners, how it evolved across time).

Giving Feedback to Peers

The next questions ask about when you gave feedback to peers across the course.

11. It was hard to deliver constructive criticism to my *1st peer partner* honestly. *

Mark only one oval.

- 0 - Not at all true
- 1 - Somewhat true
- 2 - Moderately true
- 3 - Very true
- 4 - Completely true

12. It was hard to deliver constructive criticism to my *2nd peer partner* honestly. *

Mark only one oval.

- 0 - Not at all true
- 1 - Somewhat true
- 2 - Moderately true
- 3 - Very true
- 4 - Completely true

13. Sometimes with my *1st peer partner*, my constructive feedback towards didn't show how I really felt inside. *

Mark only one oval.

- 0 - Not at all true
- 1 - Somewhat true
- 2 - Moderately true
- 3 - Very true
- 4 - Completely true

14. Sometimes with my *2nd peer partner*, my constructive feedback towards didn't show how I really felt inside. *

Mark only one oval.

- 0 - Not at all true
- 1 - Somewhat true
- 2 - Moderately true
- 3 - Very true
- 4 - Completely true

15. Please elaborate on your experience giving constructive feedback to your peer roleplay partners (e.g., how it compared between partners, how it evolved across time). *

16. Mark the importance of the following factors for making feedback exchange effective? *

Mark only one oval per row.

	0 - not at all important	1	2	3	4	5	6	7 - extreme importa
**Openness to feedback**	◯	◯	◯	◯	◯	◯	◯	◯
**Delivering feedback with kindness**	◯	◯	◯	◯	◯	◯	◯	◯
**Feedback that focuses on specific therapy skills**	◯	◯	◯	◯	◯	◯	◯	◯
**Feedback that focuses on how it made the client feel**	◯	◯	◯	◯	◯	◯	◯	◯
**Feedback that focuses on therapist style/approach**	◯	◯	◯	◯	◯	◯	◯	◯
**Explicit discussion about feedback expectations**	◯	◯	◯	◯	◯	◯	◯	◯
**Established trust over time**	◯	◯	◯	◯	◯	◯	◯	◯
**Balancing positive and constructive feedback**	◯	◯	◯	◯	◯	◯	◯	◯

Reflecting on Feedback from Peers and AI

For the following questions, reflect on the feedback you received as a therapist in the past two outside-of-class roleplay assignments

17. To what extent did you find the feedback from your *1st peer partner* to be constructive and helpful? *

Mark only one oval.

- 0 - Not at all
- 1 - To a small extent
- 2 - To a moderate extent
- 3 - To a great extent
- 4 - To a very great extent

18. To what extent did you find the feedback from your *2nd peer partner* to be constructive and helpful? *

Mark only one oval.

- 0 - Not at all
- 1 - To a small extent
- 2 - To a moderate extent
- 3 - To a great extent
- 4 - To a very great extent

19. To what extent did you find the AI feedback to be constructive and helpful? *

Mark only one oval.

- 0 - Not at all
- 1 - To a small extent
- 2 - To a moderate extent
- 3 - To a great extent
- 4 - To a very great extent

20. I felt comfortable receiving feedback from my *1st peer partner* *

Mark only one oval.

- Disagree Strongly
- Disagree Moderately
- Disagree Slightly
- Agree Slightly
- Agree Moderately
- Agree Strongly

21. I felt comfortable receiving feedback from my *2nd peer partner* *

Mark only one oval.

- Disagree Strongly
- Disagree Moderately
- Disagree Slightly
- Agree Slightly
- Agree Moderately
- Agree Strongly

22. The feedback from my *1st peer partner* helped me recognize specific areas where I can improve *

Mark only one oval.

- Disagree Strongly
- Disagree Moderately
- Disagree Slightly
- Agree Slightly
- Agree Moderately
- Agree Strongly

23. The feedback from my *2nd peer partner* helped me recognize specific areas where I can improve *

Mark only one oval.

- Disagree Strongly
- Disagree Moderately
- Disagree Slightly
- Agree Slightly
- Agree Moderately
- Agree Strongly

24. The AI feedback has helped me recognize specific areas where I can improve. *

Mark only one oval.

- Disagree Strongly
- Disagree Moderately
- Disagree Slightly
- Agree Slightly
- Agree Moderately
- Agree Strongly

25. Please elaborate on your experience receiving feedback from your role play partners (e.g., how it compared between partners, how it evolved across time).

26. Please elaborate on your experience receiving AI feedback (e.g., helping your learning, how its use or utility has evolved over time)

27. Were there any weeks where the peer feedback discussion was particularly helpful/insightful?

28. Were there any weeks where AI feedback was particularly helpful/insightful?

Partner's AI Feedback (2/4)

Think back to the roleplay sessions where you were the client and your partner was the therapist, and they received AI feedback on their therapy skills.

29. How often did you review the AI feedback given on your partner's therapy roleplay? *

Mark only one oval.

- I never reviewed the AI feedback on their therapy performance.
- I reviewed the AI feedback for some of the weeks.
- I reviewed the AI feedback every week.
- I reviewed the AI feedback multiple times each week.

30. To what extent was the AI feedback your partner received helpful for your own learning? *

Mark only one oval.

- N/A - I did not view the AI feedback for transcripts where my partner was the therapist
- 1 - Not at all
- 2 - To a small extent
- 3 - To a moderate extent
- 4 - To a great extent
- 5 - To a very great extent

31. Reviewing the AI feedback given to my partner led me to adjust my use of therapy skills. *

Mark only one oval.

- N/A - I did not view the AI feedback for transcripts where my partner was the therapist
- Disagree Strongly
- Disagree Moderately
- Disagree Slightly
- Agree Slightly
- Agree Moderately
- Agree Strongly

32. Please elaborate on any notable learning that occurred from reviewing feedback about your partner's therapist roleplay.

____

____

____

____

____

Constructive Feedback Conversations (3/4)

The next questions ask you about the constructive feedback conversations you have had with your partner in the 1st half of the course

33. It was hard to deliver constructive criticism to my peer honestly. *

Mark only one oval.

- 0 - Not at all true
- 1 - Somewhat true
- 2 - Moderately true
- 3 - Very true
- 4 - Completely true

34. Sometimes, my constructive feedback didn't show how I really felt inside. *

Mark only one oval.

- 0 - Not at all true
- 1 - Somewhat true
- 2 - Moderately true
- 3 - Very true
- 4 - Completely true

35. Please elaborate on your experience giving constructive feedback to your peer roleplay partners (e.g., how it compared across partners, how it evolved over time) *

36. Mark the importance of the following factors for making feedback exchange effective? *

Mark only one oval per row.

	0 - not at all important	1	2	3	4	5	6	7 - extreme importa
**Openness to feedback**								
**Delivering feedback with kindness**								
**Feedback that focuses on specific therapy skills**								
**Feedback that focuses on how it made the client feel**								
**Feedback that focuses on therapist style/approach**								
**Joint review of AI feedback**								
**Explicit discussion about feedback expectations**								
**Established trust over time**								
**Balancing positive and constructive feedback**								

AI Feedback Influence (4/4)

The next questions ask you how AI feedback has influenced the feedback discussions with your partner

37. To what extent has receiving or viewing others receiving AI feedback changed how you deliver feedback to your peers? *

Mark only one oval.

- 1 - Not at all
- 2 - To a small extent
- 3 - To a moderate extent
- 4 - To a great extent
- 5 - To a very great extent

38. How has the availability of AI feedback affected your comfort level in giving constructive criticism to your peers? *

Mark only one oval.

- Much less comfortable
- Somewhat less comfortable
- No change in comfort level
- Somewhat more comfortable
- Much more comfortable

39. Please elaborate whether AI feedback has influenced how you exchange feedback with your partner, and how it has evolved over time. *

This content is neither created nor endorsed by Google.

Google Forms
