# Supplementary material for: Classroom Integration of AI-Simulated Patients and Automated Feedback in Doctoral Psychotherapy Training: Longitudinal Mixed Methods Study
Source: Interact J Med Res. 2026 Nov 11;15:e93199. doi: 10.2196/93199 (PMC13754175; doi:10.2196/93199)
Supplement: Supplementary file 4 [file ijmr-v15-e93199-s004.pdf]

Stanford

## Introduction

We would like to learn about your experiences using **CARE** and **Skillsetter** in CLIN 702: Learning Psychotherapy.

## Email

Enter the email address you use to login to **CARE** (e.g., name@care.edu)

## Overall

Artificial intelligence (AI) is designed to learn tasks that humans typically do, for instance recognizing speech or pictures.

How much have you heard or read about AI?

| | 0 | 10 | 20 | 30 | 40 | 50 | 60 | 70 | 80 | 90 | 100 |
|---|---|---|---|---|---|---|---|---|---|---|---|
| (0 - not at all familiar, 50 - somewhat familiar, 100 - extremely familiar) | | | | | | | | | | | |

Overall, would you say the increased use of artificial intelligence (AI) in daily life makes you feel...

- More concerned than excited
- Equally excited and concerned
- More excited than concerned

How much do you use AI in other aspects of your personal or school life?

| | 0 | 10 | 20 | 30 | 40 | 50 | 60 | 70 | 80 | 90 | 100 |
|---|---|---|---|---|---|---|---|---|---|---|---|
| (0 - never use, 50 - sometimes use, 100 - use all the time) | | | | | | | | | | | |

Do you think artificial intelligence (AI) in the classroom would lead to better or worse helping skills for therapists?

- Better
- Worse
- No Change

How has the course---from about CARE AI's role in the class, or assigned readings about AI in psychotherapy---influenced your thinking about AI's use in psychotherapy education and practice?

Once **CARE** became optional, how did you make your decision whether you'd continue using it for deliberate practice?

Now that you have experienced **CARE** AI, would you recommend next year's cohort of PsyD students use **CARE** AI as one of the deliberate practice options?

- No, because:
- Yes, because:
- Yes, but only if the following changes were made:

For the following questions you'll be able to select **concerns** that were top of mind.

My concerns about the functionality of **CARE** AI's **patients** include (select all that apply).

- ☐ Does not recognize my voice
- ☐ AI patients do not feel realistic
- ☐ Voices sound robotic
- ☐ The transcript of the audio-interaction is often inaccurate
- ☐ The emotional expression of voices were limited
- ☐ Not detecting vocal pauses
- ☐ No visual avatar
- ☐ Other concerns

My concerns about **CARE** AI's **feedback** include (select all that apply).

- ☐ AI providing unhelpful feedback
- ☐ I wish it gave feedback on my speech, rather than just the written transcript of what I said.
- ☐ I wish its evaluation criteria had more variety each week
- ☐ I wish it gave feedback on the session as a whole
- ☐ I wish CARE's feedback system tracked and referenced how I was progressing over time
- ☐ I wish the AI patient could give me feedback from their perspective on how I made them feel
- ☐ Other concerns

My concerns about how **CARE** was **integrated into the classroom** experience include (select all that apply).

- ☐ How my data is used by the research team
- ☐ AI replacing peer interaction
- ☐ AI replacing peer support and feedback
- ☐ Environmental impact
- ☐ I feel that my data is going to be used to make an AI therapist
- ☐ What if they are making an AI therapist to replace me

- ☐ Am I just doing user testing for a product
- ☐ Other concerns:

**Practice Format**

On this page, you will be asked to compare your experience **practicing responding to patients** with CARE vs. Skillsetter

The way I could use **CARE** allowed me to make mistakes that I could learn from.

- ◯ Disagree Strongly
- ◯ Disagree Moderately
- ◯ Disagree Slightly
- ◯ Agree Slightly
- ◯ Agree Moderately
- ◯ Agree Strongly

Recording myself responding to video vignettes in **Skillsetter** allowed me to make mistakes that I could learn from.

- Disagree Strongly
- Disagree Moderately
- Disagree Slightly
- Agree Slightly
- Agree Moderately
- Agree Strongly

It was easy to immerse myself in the therapist/patient interaction in **CARE**.

- Disagree Strongly
- Disagree Moderately
- Disagree Slightly
- Agree Slightly
- Agree Moderately
- Agree Strongly

It was easy to immerse myself in the therapist/patient interaction in **Skillsetter**.

- Disagree Strongly
- Disagree Moderately
- Disagree Slightly
- Agree Slightly
- Agree Moderately
- Agree Strongly

In **CARE**, I could practice important aspects of therapeutic communication beyond just choosing the right words (e.g., tone of voice, pacing, pausing, voice modulation).

- Disagree Strongly
- Disagree Moderately
- Disagree Slightly
- Agree Slightly
- Agree Moderately
- Agree Strongly

In **Skillsetter**, I could practice important aspects of therapeutic communication beyond just choosing the right words (e.g., tone of voice, pacing, pausing, voice modulation).

- Disagree Strongly
- Disagree Moderately
- Disagree Slightly
- Agree Slightly
- Agree Moderately
- Agree Strongly

**CARE** prepares me to feel comfortable/confident thinking on my feet with a patient (i.e., responding spontaneously).

- Disagree Strongly
- Disagree Moderately
- Disagree Slightly
- Agree Slightly
- Agree Moderately
- Agree Strongly

**Skillsetter** prepares me to feel comfortable/confident thinking on my feet with a patient (i.e., responding spontaneously).

- ○ Disagree Strongly
- ○ Disagree Moderately
- ○ Disagree Slightly
- ○ Agree Slightly
- ○ Agree Moderately
- ○ Agree Strongly

After conversing with the AI patients in **CARE** I'm more equipped to build and maintain a therapeutic alliance.

- ○ Disagree Strongly
- ○ Disagree Moderately
- ○ Disagree Slightly
- ○ Agree Slightly
- ○ Agree Moderately
- ○ Agree Strongly

After video recording myself responding to the patient in **Skillsetter** I'm more equipped to build and maintain a therapeutic alliance.

- ◯ Disagree Strongly
- ◯ Disagree Moderately
- ◯ Disagree Slightly
- ◯ Agree Slightly
- ◯ Agree Moderately
- ◯ Agree Strongly

If you have any other thoughts, comparisons, or suggestions regarding the **Skillsetter's** and **CARE's** AI patient practice, please elaborate on them here:

## Feedback

On this page, you will be asked about your experience **receiving feedback** in the course.

Receiving AI feedback shortly after completing the **CARE** practice enhanced my learning experience.

- ◯ Disagree Strongly
- ◯ Disagree Moderately
- ◯ Disagree Slightly
- ◯ Agree Slightly
- ◯ Agree Moderately
- ◯ Agree Strongly

Unlike waiting days for teaching team feedback, receiving AI feedback moments after **CARE** practice enhanced my retention of key learning points.

- ◯ Disagree Strongly
- ◯ Disagree Moderately

- Disagree Slightly
- Agree Slightly
- Agree Moderately
- Agree Strongly

The AI feedback system has the necessary skills and expertise to provide accurate feedback on my work.

- Disagree Strongly
- Disagree Moderately
- Disagree Slightly
- Agree Slightly
- Agree Moderately
- Agree Strongly

I know when to ignore the AI feedback when it is incorrect or does not make sense.

- Disagree Strongly
- Disagree Moderately
- Disagree Slightly

- ◯ Agree Slightly
- ◯ Agree Moderately
- ◯ Agree Strongly

If you have any other comparisons or suggestions about how feedback was delivered—from **CARE's** AI feedback system and/or the teaching team—please elaborate on them here:

**Reflection**

I found the self-reflections after a **CARE** session valuable for understanding how to improve.

- ◯ Disagree Strongly
- ◯ Disagree Moderately
- ◯ Disagree Slightly
- ◯ Agree Slightly
- ◯ Agree Moderately

- Agree Strongly

I found the self-reflections after the **Skillsetter** recording valuable for understanding how to improve.

- Disagree Strongly
- Disagree Moderately
- Disagree Slightly
- Agree Slightly
- Agree Moderately
- Agree Strongly

If you continued using **CARE** in the second part of the course, you were provided with various types of self-reflection experiences for each new practice session.

In this section, we'll (1) show a screenshot of the specific self-reflection experience; and (2) ask you to reflect on the components.

Utterance-Level Self-Reflection Questions, without AI feedback

**1. What was your intention behind your response?**

Enter your intentions...

**2. How do you think this may have impacted the way the patient next responded?**

Describe how it led to the patient's reply...

**3. Based on your reflection, would you keep or modify your response? If modifying, please write your new response.**

Describe how you might respond differently...

Submit Reflection

Did you get an opportunity to receive these utterance-level self-reflection questions?

- Yes
- No
- I don't know

What if anything did you like or find useful about these utterance-level self-reflection questions?

- ☐ I liked understanding the impact my words had on the AI patients response
- ☐ I liked getting a second chance to modify my response
- ☐ [ ] Other:

Things you disliked or wish were different about utterance-level self-reflection questions?

- ☐ There were too many of them to answer
- ☐ The standard reflections become repetitive and boring.
- ☐ I prefer to reflect on my overall approach to a practice session
- ☐ I wish I knew why some responses were chosen to reflect upon
- ☐ I wish I could choose which utterances to reflect on
- ☐ [ ] Other dislikes/limitations:

# AI Feedback with Utterance-Level Self-Reflections

Strengths Questions Feedback Areas Validation Empathy

Feedback The goal is to empathize with the seeker's feelings of being left out. Instead of immediately asking for more details, it would be more effective to validate their feelings and offer empathy.

Alternate Response

It sounds like you've been through a lot. It's really tough when you feel like you're the one dealing with the aftermath of your family's disapproval. How are you dealing with this?

**1. What are the important differences between your response and the alternate response?**

Enter the important differences...

**2. Would the patient have responded differently to the alternate response, and if yes, do you think that would have been helpful and why?**

Describe how the patient might have responded differently to the alternate response...

**3. Having reflected on both responses, please write out how you would respond now if you had another opportunity with this patient.**

Describe how you might respond differently...

Submit Reflection

Did you get an opportunity to receive AI feedback plus utterance-level self-reflection questions?

- ◯ Yes
- ◯ No
- ◯ I don't know

What if anything did you like or find useful about these utterance self-reflection questions?

- I liked processing my thoughts on the AI feedback by reflecting
- I liked that I could critically engage with the AI feedback, rather than accept it as the gold standard
- I liked understanding the impact that one's words could have on an AI patients response
- I liked getting a second chance to modify my response
- [ ] Other likes/benefits:

Things you disliked or wish were different about AI feedback with utterance-level self-reflection questions?

- There were too many of them to answer
- The standard reflections become repetitive and boring.
- I prefer to reflect on my overall approach to a practice session
- I wish I knew why some responses were chosen to reflect upon
- I wish I could choose which utterances to reflect on
- [ ] Other dislikes/limitations:

Powered by Qualtrics
