# Supplementary material for: Classroom Integration of AI-Simulated Patients and Automated Feedback in Doctoral Psychotherapy Training: Longitudinal Mixed Methods Study
Source: Interact J Med Res. 2026 Nov 11;15:e93199. doi: 10.2196/93199 (PMC13754175; doi:10.2196/93199)

CARE AI FAQ

Last updated: 10-25-2024

Content

1. Why did I not receive a consent form to sign?
2. If I didn't sign a consent form, does this mean that I'm being forced to participate?
3. Who are the researchers that created CARE?
4. How can I learn more about related AI research?
5. Who can I ask questions to about CARE?
6. What if CARE is not capturing my audio accurately?
7. What exactly is the plan of using the transcripts?
8. Why am I not getting a confirmation email when I register for CARE?

1. Why did I not receive a consent form to sign?

- The CARE AI tool is included in the curriculum for the course, Learning Psychotherapy (CLIN 701). Much like the roleplay aspect in class, the CARE AI feedback tool is part of the class this year too.

- This type of research study is called a “Normal Educational Practice” (IRB Social and Behavioral Sciences (IRB-SBS), a category that often applies to educational studies
- For more information on normal educational practice, see Normal Educational Practice.

2. If I didn’t sign a consent form, does this mean that I’m being forced to participate?

- As a student in CLIN 701, you are not being forced to use CARE. The option to opt-in to using this component of the class is available for everyone. Opting out does not impact your grade for CLIN 701 in any way.

3. Who are the researchers that created CARE?

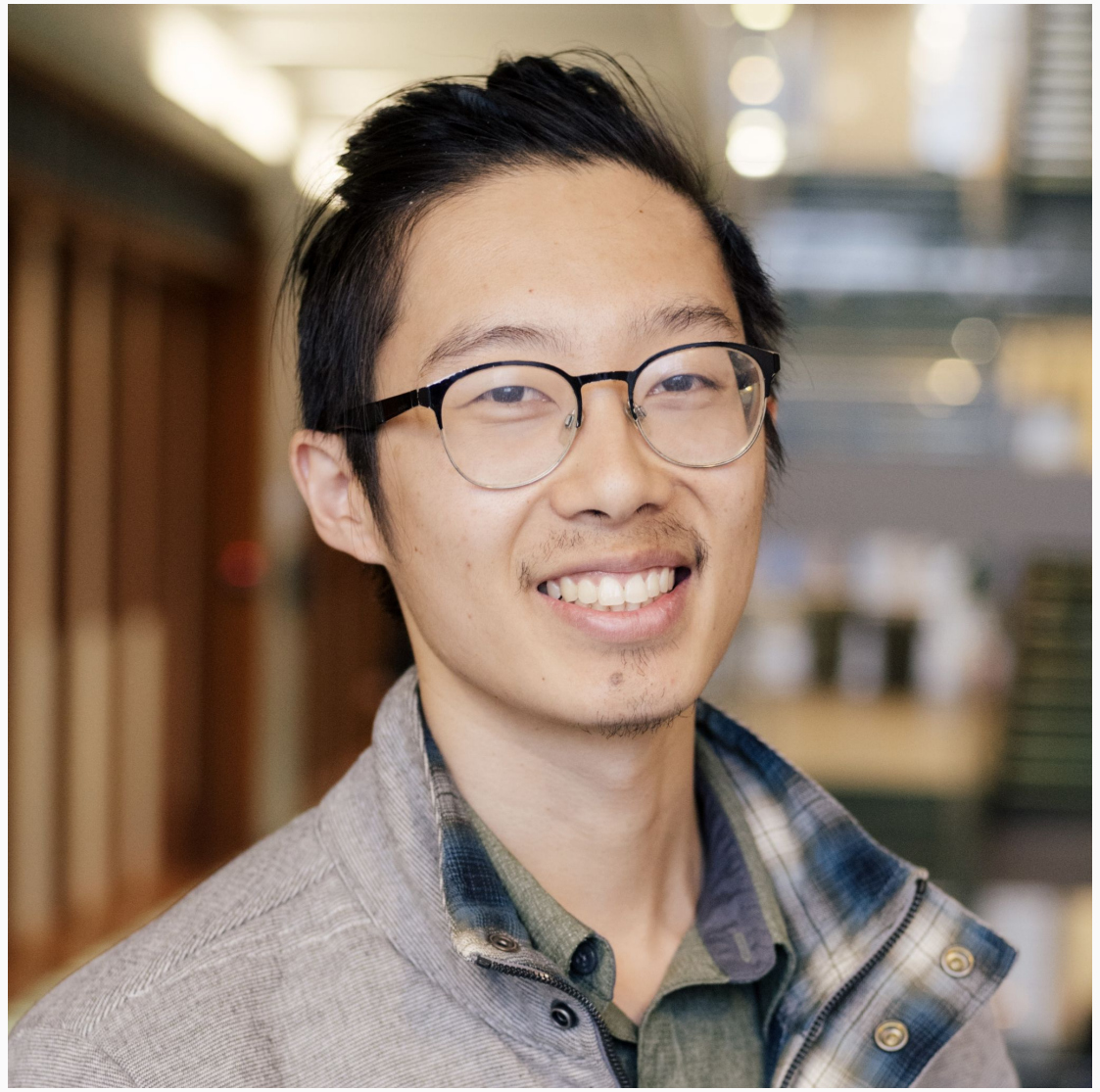

Ryan Louie: I am a Postdoctoral Researcher at Stanford University CS, where I collaborate with Diyi Yang and Emma Brunskill to advance AIs potential to assist in Mental Health and Psychotherapy. Mental health providers--from trained therapists to volunteer peer supporters--remain crucial to mental health care, and we hypothesize that AI-assisted training and practice can improve their efficacy, confidence, and growth. Human-AI collaboration and interfaces for effectively imbuing AIs with domain-specific and personal-knowledge of this area will be important.

3. Who are the researchers that created CARE?

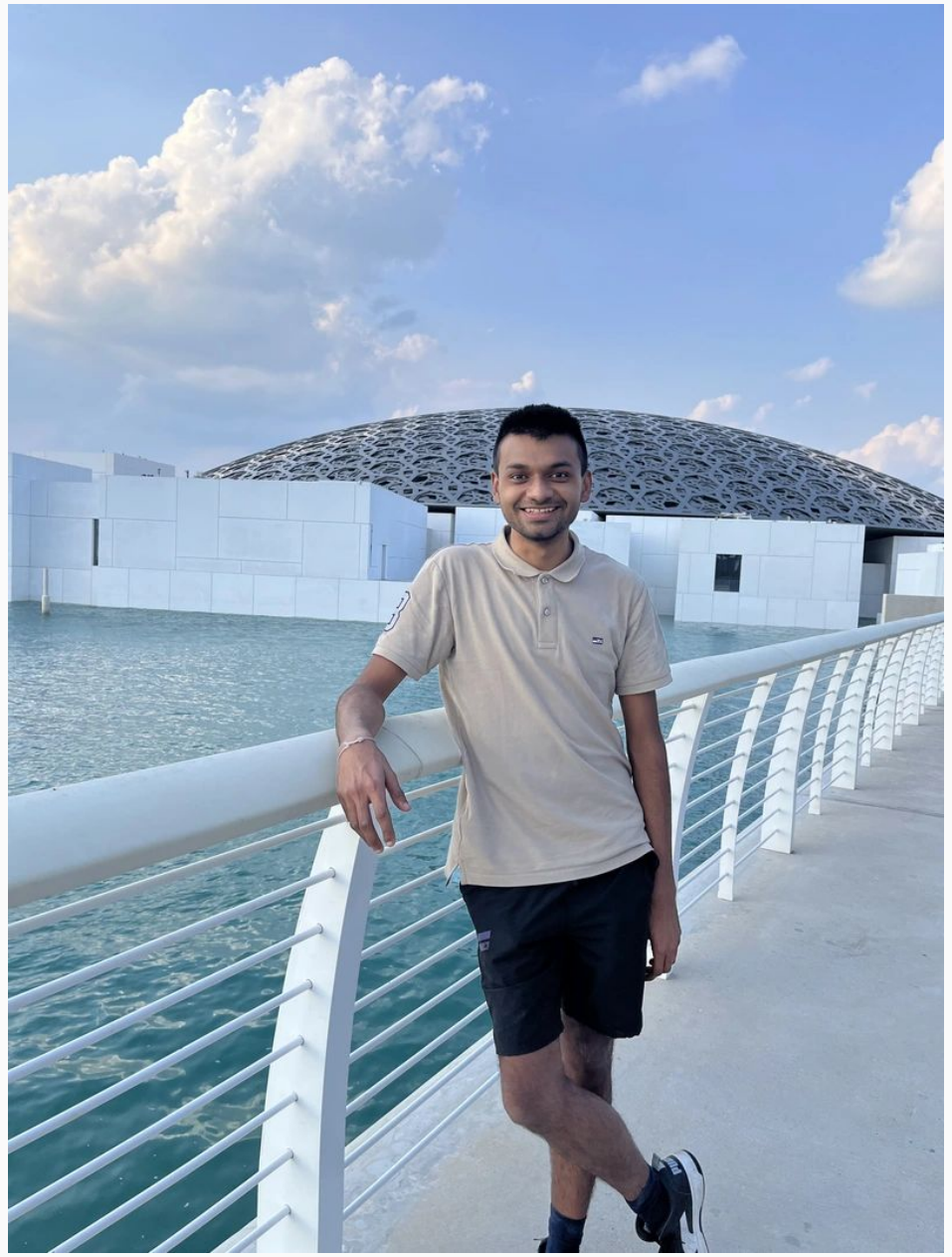

Raj Sanjay Shah: I am a PhD student at Interactive Computing, Georgia Tech, and currently a visiting researcher with Prof. Diyi Yang at Stanford. I am interested in building human-AI collaborative natural language processing models for social good. My previous research looks at using tools to understand online peer support platforms and determine the key skills for peers to practice and provide effective support. In the longer term, I wish to develop technologies and tools that help communication and educational practices of therapists, ones that augment human skills.

3. Who are the researchers that created CARE?

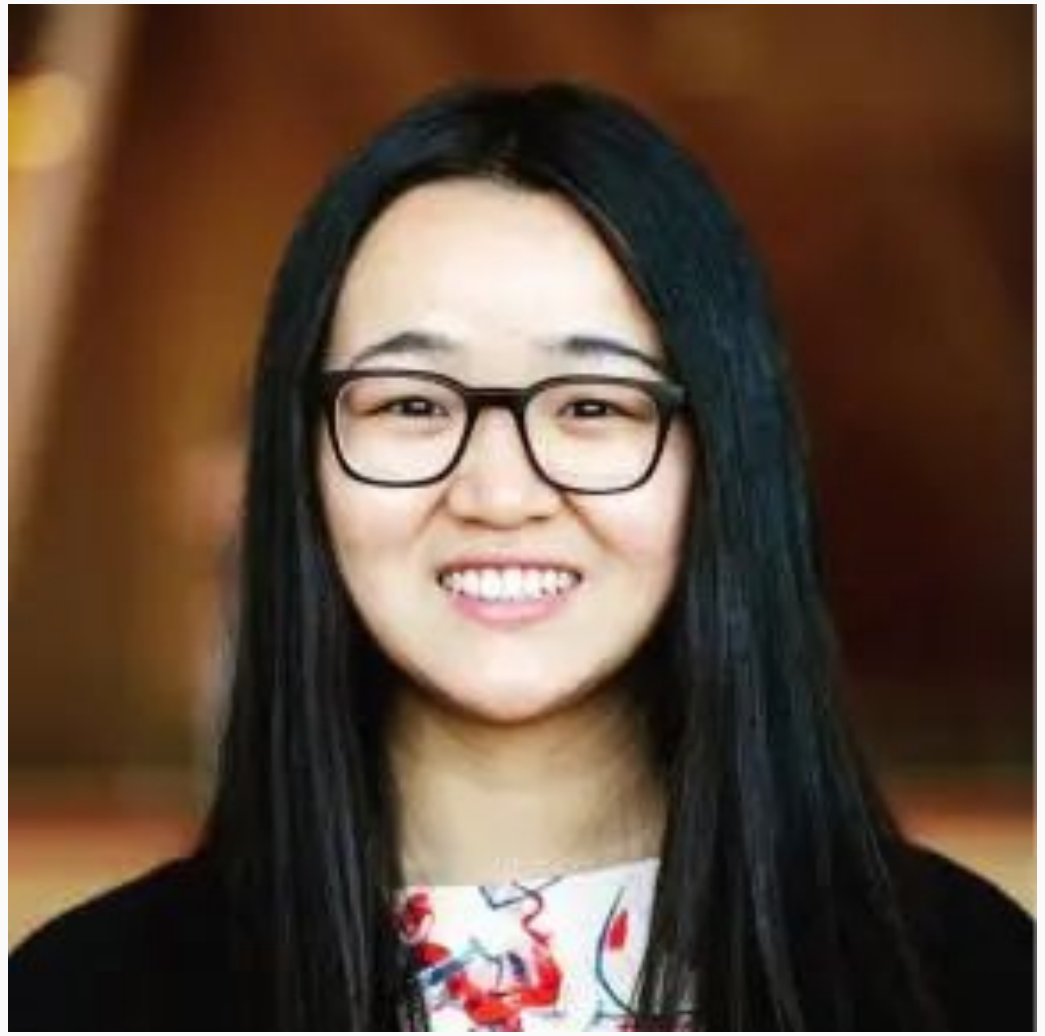

Diyi Yang: I am an assistant professor in the Computer Science Department at Stanford, affiliated with the NLP Group, HCI Group, Stanford AI Lab (SAIL), and Stanford Human-Centered Artificial Intelligence (HAI). I am interested in Computational Social Science, and Natural Language Processing. My research goal is to better understand human communication in social context and build socially aware language technologies to support human-human and human-computer interaction.

3. Who are the researchers that created CARE?

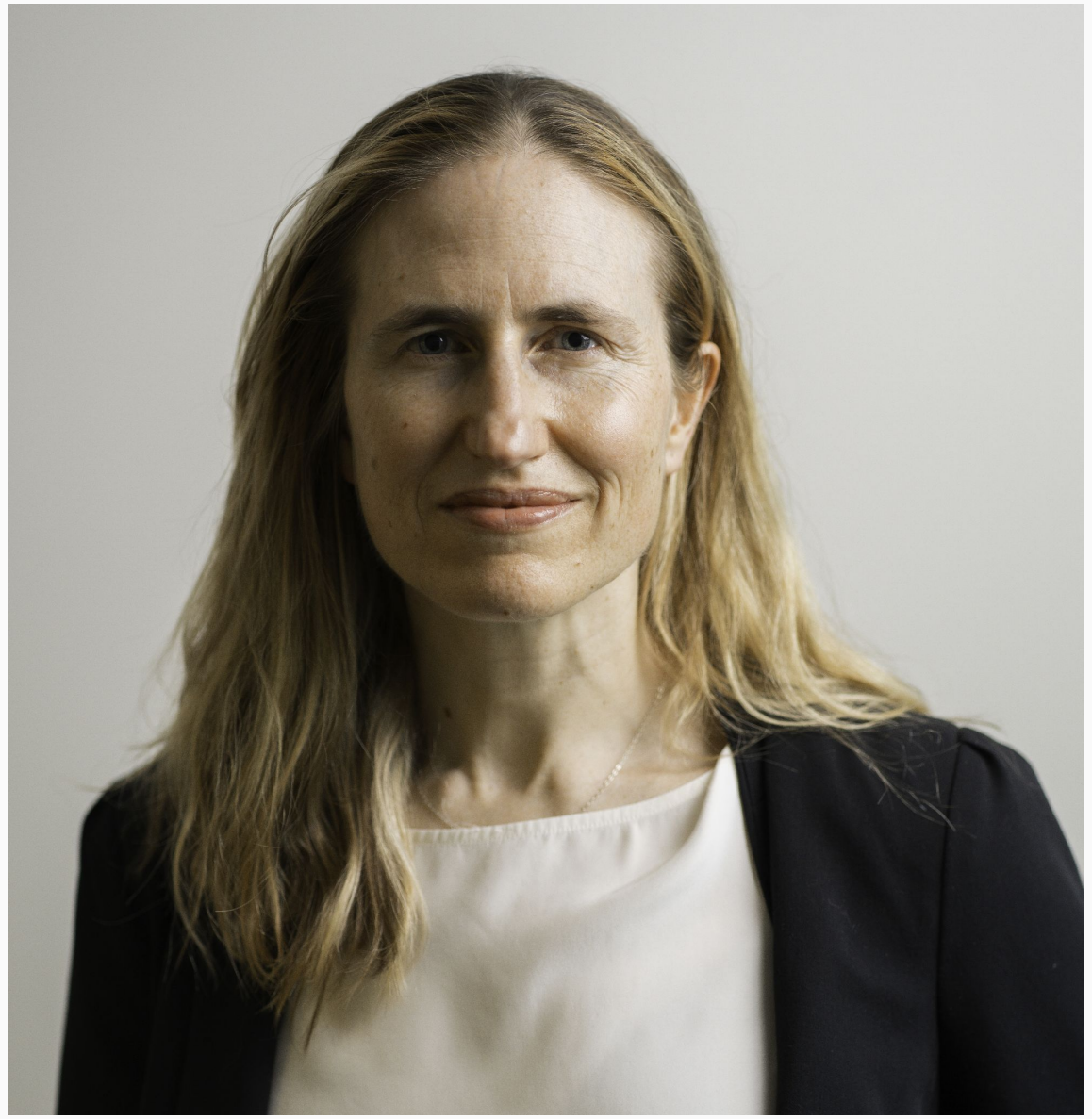

Emma Brunskill: I am an associate tenured professor in the Computer Science Department at Stanford University, a principal investigator on the Empowering Peer Supporters team (a Stage 1 impact lab), and a 2023 Scholar in Service working with Carnegie Learning to leverage AI to improve middle school math educational outcomes. My goal is to create AI systems that learn from few samples to robustly make good decisions, motivated by our applications to healthcare and education. I am privileged to serve on the International Machine Learning Society (which coordinates ICML) Board, the Khan Academy Research Advisory Board, the Stanford Faculty Women's Forum Steering Committee, and I previously served on the Women in Machine Learning (WIML) board.

4. How can I learn more about the team's human-centered AI research?

Human-Centered AI Research and AI for Social Impact:

- Read about our Stanford Impact Lab Grant that speaks to a parallel vision in using AI to empower novice supporters
- Read on Stanford's Human AI blog about the backstory behind CARE's AI feedback tool:
- Diyi Yang gave a talk at the Computer History Museum: Human-AI Interactions in the Age of LLMs. Professor Diyi Yang (AE Spring Summit 2024).

5. Who can I ask questions to about CARE?

There are a few options.

- You can describe your experience anonymously in the mid-class survey
- If can raise questions anonymously in a question form
- You can let Ellen know if you have any questions. If she doesn’t know the answer, she will connect with Ryan/Raj over her weekly meetings with them, or via email.

6. What if CARE is not capturing my audio accurately?

If you can, try to ensure that your environment is quiet. Background noise can negatively impact the transcription process.

7. Why am I not getting a confirmation email when I register for CARE?

The purpose of entering the “email” is to create an identifier linking your transcript to you. While the format looks like an email, this is merely an identifier and you could technically put anything in the “email box” (example: your first name, a nickname, some numbers).

8. What exactly is happening with my transcripts?

The video-transcripts are being stored on a secure password-protected server in the Cloud. If there are audio upload/transcription issues, Ryan has logged into to that server to understand what file was uploaded that is causing it. Besides this case, we are not using the audio files for anything else and they will be deleted after the research analysis of the study is finished.

The text transcripts are stored on CARE's database, stored on the secure password-protected server. These transcripts are viewable by you and the partner you've selected. If you choose the "Review Feedback tool" the transcript is processed by our AI feedback model. This model is self-hosted, meaning we are not using commercial products like ChatGPT to process. This ensures your transcript data is not used by these companies to train their next version of an AI model.

The information sheet details all things we're committed to in this study protocol, and which is verified by the Stanford IRB.

8. What exactly is happening with my transcripts?

Data Control of Specific SEssions: You can let us know about specific transcripts that you would like to delete or have Ryan/Raj exclude from their analysis. Please send us an email of the URL of the chat transcript so it will be added to an **exclude list**

URL can be found at the top of the page

https://care.ngrok.app/chat/311db75358d34e6096acf5ef83e19567/Ellen&listener

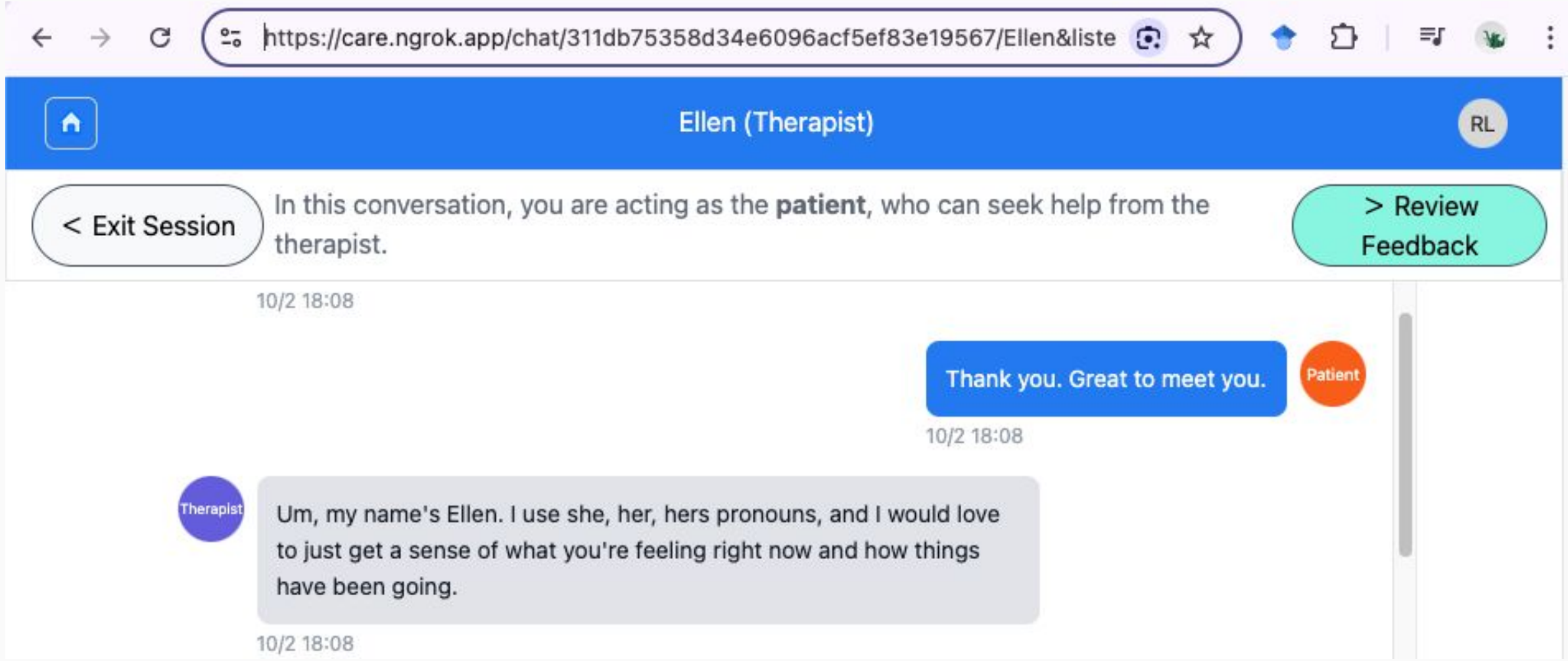

9. Can CARE provide MI-specific feedback?

CARE's feedback model uses **8 skills** that overlap between the Helping Skills and MI model [link to paper]

Thus, it does not provide MI-specific feedback.

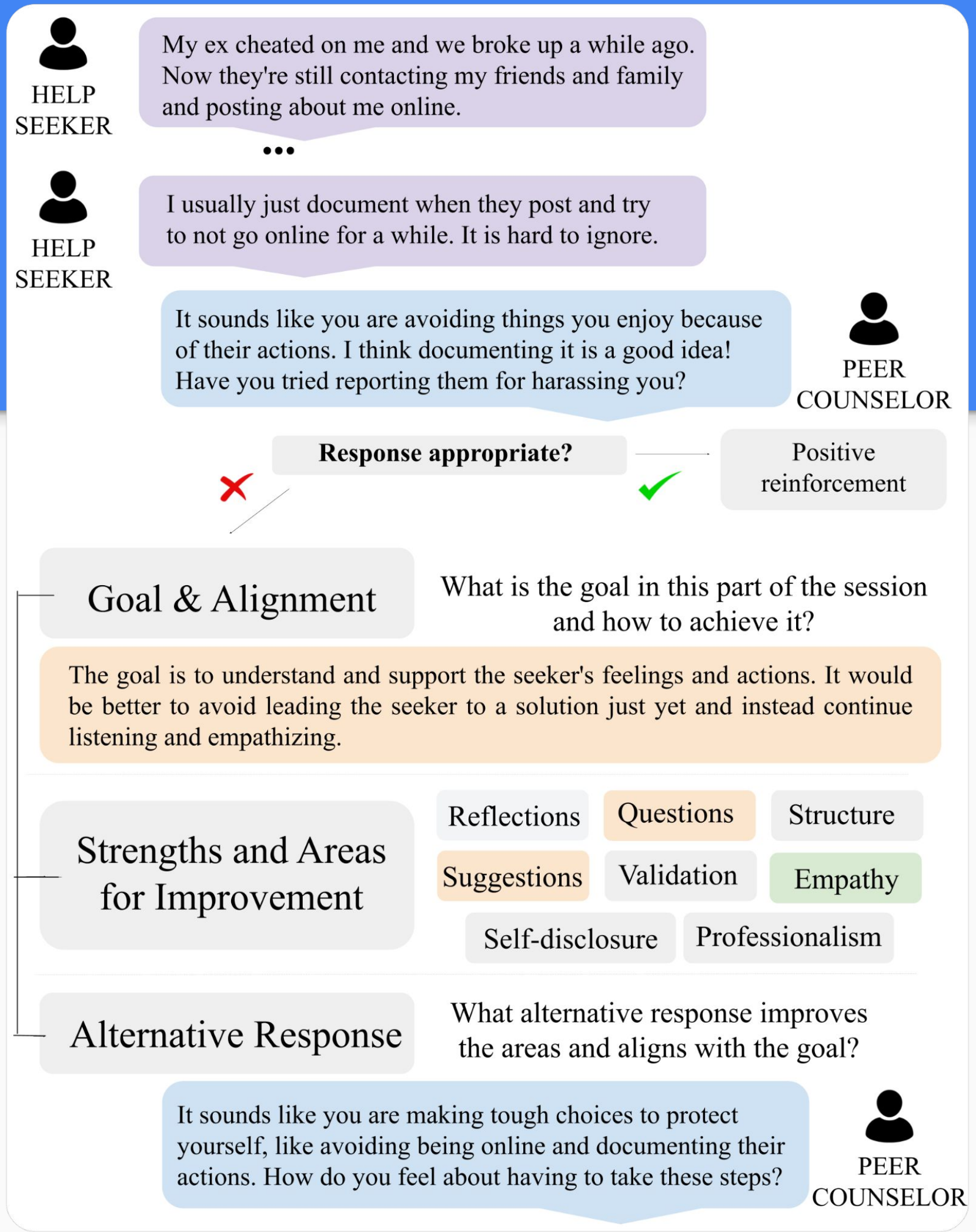

Supplement: Supplementary file 5 [file ijmr-v15-e93199-s005.pdf]
